# A Ubiquitin-specific Protease Possesses a Decisive Role for Adenovirus Replication and Oncogene-mediated Transformation

**DOI:** 10.1371/journal.ppat.1003273

**Published:** 2013-03-28

**Authors:** Wilhelm Ching, Emre Koyuncu, Sonia Singh, Christina Arbelo-Roman, Barbara Hartl, Elisabeth Kremmer, Thomas Speiseder, Chris Meier, Thomas Dobner

**Affiliations:** 1 Department of Molecular Virology, Heinrich Pette Institute, Leibniz Institute for Experimental Virology, Hamburg, Germany; 2 Organic Chemistry, Department of Chemistry, Faculty of Sciences, University of Hamburg, Hamburg, Germany; 3 Institute for Molecular Immunology, Helmholtz Centre Munich, Munich, Germany; Wake Forest University, United States of America

## Abstract

Adenoviral replication depends on viral as well as cellular proteins. However, little is known about cellular proteins promoting adenoviral replication. In our screens to identify such proteins, we discovered a cellular component of the ubiquitin proteasome pathway interacting with the central regulator of adenoviral replication. Our binding assays mapped a specific interaction between the N-terminal domains of both viral E1B-55K and USP7, a deubiquitinating enzyme. RNA interference-mediated downregulation of USP7 severely reduced E1B-55K protein levels, but more importantly negatively affected adenoviral replication. We also succeeded in resynthesizing an inhibitor of USP7, which like the knockdown background reduced adenoviral replication. Further assays revealed that not only adenoviral growth, but also adenoviral oncogene-driven cellular transformation relies on the functions of USP7. Our data provide insights into an intricate mechanistic pathway usurped by an adenovirus to promote its replication and oncogenic functions, and at the same time open up possibilities for new antiviral strategies.

## Author Summary

Adenoviral infections can result in severe outcomes leading to mortality especially in children undergoing immunosuppressive therapies. Unfortunately, no specific anti-adenoviral treatments are available to treat disseminated adenoviral infections. We have set out to identify host factors promoting adenoviral growth and could identify the cellular protein Ubiquitin-specific protease 7 (USP7) being central to adenoviral infection. Here we show that USP7 interacts with the viral protein E1B-55K, a central regulator of adenoviral replication and adenoviral oncogene-mediated cellular transformation. We demonstrate that USP7 ensures stability and/or proper expression levels of adenoviral proteins at early and late time points of infection. Consistent with this, small-molecule inhibitors of USP7 showed efficient reduction of capsid protein levels and viral progeny numbers. Thus, USP7 inhibition might be a useful treatment option in the context of disseminated adenoviral infections. Moreover, we were also able to show that adenoviral oncogene-mediated cellular transformation can be hampered by USP7 disruption. In summary, this study shows that two different adenoviral disease mechanisms can be inhibited by targeting one host cellular factor.

## Introduction

Human adenoviruses constitute a group of more than 60 adenovirus types. In general, adenoviruses cause self-limiting infections of the eye, or gastrointestinal and respiratory tract, which can lead to epidemic keratoconjunctivitis, diarreah, and severe acute respiratory diseases [1]–[9]. However, with increasing prevalence of transplantations with concomittant downregulation of the immune system (such as in bone marrow transplations), the frequency of disseminated adenoviral infections is also rising in immuno-compromised patients, resulting in high mortality rates [10], [11]. Unfortunately, no specified antiviral treatments or wide-spread vaccination strategies are currently available to counteract adenoviral outbreaks in an efficient manner [12], [13].

For successful infection, adenoviruses, like other viruses, must circumvent certain antiviral defense mechanisms. In this regard, the ubiquitin proteasome system (UPS) adopts a central position in aiding viral infections. For example, HSV-1, HPV-16/18 and EBV have been shown to use strategies which involve targeting cellular proteins with antiviral functions, such as p53, for proteasomal degradation using viral encoded or components of cellular E3 ubiquitin ligases [14]–[17]. Adenoviruses use two viral regulatory proteins, E4orf6 and E1B-55K, to exploit cellular factors to form an SCF-like E3 ubiquitin ligase complex promoting p53, Mre11, Bloom helicase (BLM), DNA ligase IV, integrin alpha 3 and Tip60 polyubiquitination followed by subsequent proteasomal degradation [18]–[23].

In contrast to all the functions involving adding ubiquitin moieties to target substrates, viral exploitation of the reverse mechanism in host cells has become increasingly important over the past few years. Deubiquitination is mediated by deubiquitinating enzymes (DUBs), and the replication of several viruses has been shown to either benefit from, or be inhibited by certain DUBs. Liao and colleagues demonstrated that Usp11 specifically inhibits influenza virus infection [24], whereas Perry and coworkers have shown that Usp14 is necessary for efficient viral replication of a panel of viruses, including norovirus, encephalomyocarditis virus, Sindbis virus, and La Crosse virus [25]. Among those DUBs, USP7 (herpesviral associated ubiquitin-specific protease [HAUSP]) was the first to be associated with viral infection, through interacting with herpesviral ICP0 [26]. Since then, more herpesviral regulatory proteins have been found to use the functions of USP7 for their own benefit. For example, EBV EBNA1 utilizes USP7's properties to stimulate its DNA-binding activity, to initiate disruption of PML proteins, to reduce p53 steady-state levels or to enhance the deubiquitination of histone H2B resulting in EBV *oriP* transcriptional activation. Furthermore, KSHV LANA probably interacts with USP7 in order to regulate latent viral genome replication [27]–[30]. Since cellular DUBs obviously represent an important family of proteins used by viral proteins, studies are underway to develop specific inhibitors of these enzymes.

Like herpesviruses, adenoviruses also encode several proteins that bind to and manipulate key cell growth regulatory proteins to promote viral replication. The adenoviral protein E1B-55K is a multifunctional phospho-protein performing central roles during productive infection, including viral mRNA transport and degradation of cellular components (e.g. p53 and Mre11), using the ubiquitin proteasome system (UPS) [21], [22]. Moreover, E1B-55K is able to induce cellular transformation of primary cells in cooperation with the adenoviral protein E1A [31], [32]. Although adenoviruses are known to be closely involved in manipulating proteins of the UPS especially through E1B-55K, to date the activity of cellular DUBs during adenoviral infection remains enigmatic and has not been studied so far.

Here, we demonstrate that the adenoviral protein E1B-55K interacts with the cellular DUB USP7. We found that USP7 is relocalized in a time-dependent manner during adenoviral infection even though independent of E1B-55K. To our interest, USP7 knockout/knockdown and inhibitor assays demonstrate that expression and/or stability of E1B-55K is strongly dependent on the presence and functions of USP7. In addition, it became evident that USP7 promotes viral growth by regulating expression and/or stability of additional adenoviral proteins. We also illustrate that adenovirus oncogene-induced transformation relies on the presence and function of USP7. Therefore, we demonstrate for the first time that general HAdV5 functions strongly depend on the availability and functions of the cellular protein USP7.

## Results

### USP7 binds to the adenovirus E1B-55K protein

E1B-55K plays key regulatory roles during adenovirus infection. This is mainly achieved through interactions with several binding partners directly or indirectly involved in p53 regulation or DNA damage response, a common strategy employed by almost all known viruses to promote viral replication and hinder antiviral defense mechanisms [33].

To discover more about the functions of E1B-55K, we profiled cellular interaction partners of E1B-55K using a yeast two-hybrid system. With the N-terminal region of HAdV5 E1B-55K protein as a bait, we identified several positive clones encoding USP7 (two positive “hit”-sequences are displayed, Figure S1).

**Figure 1:**
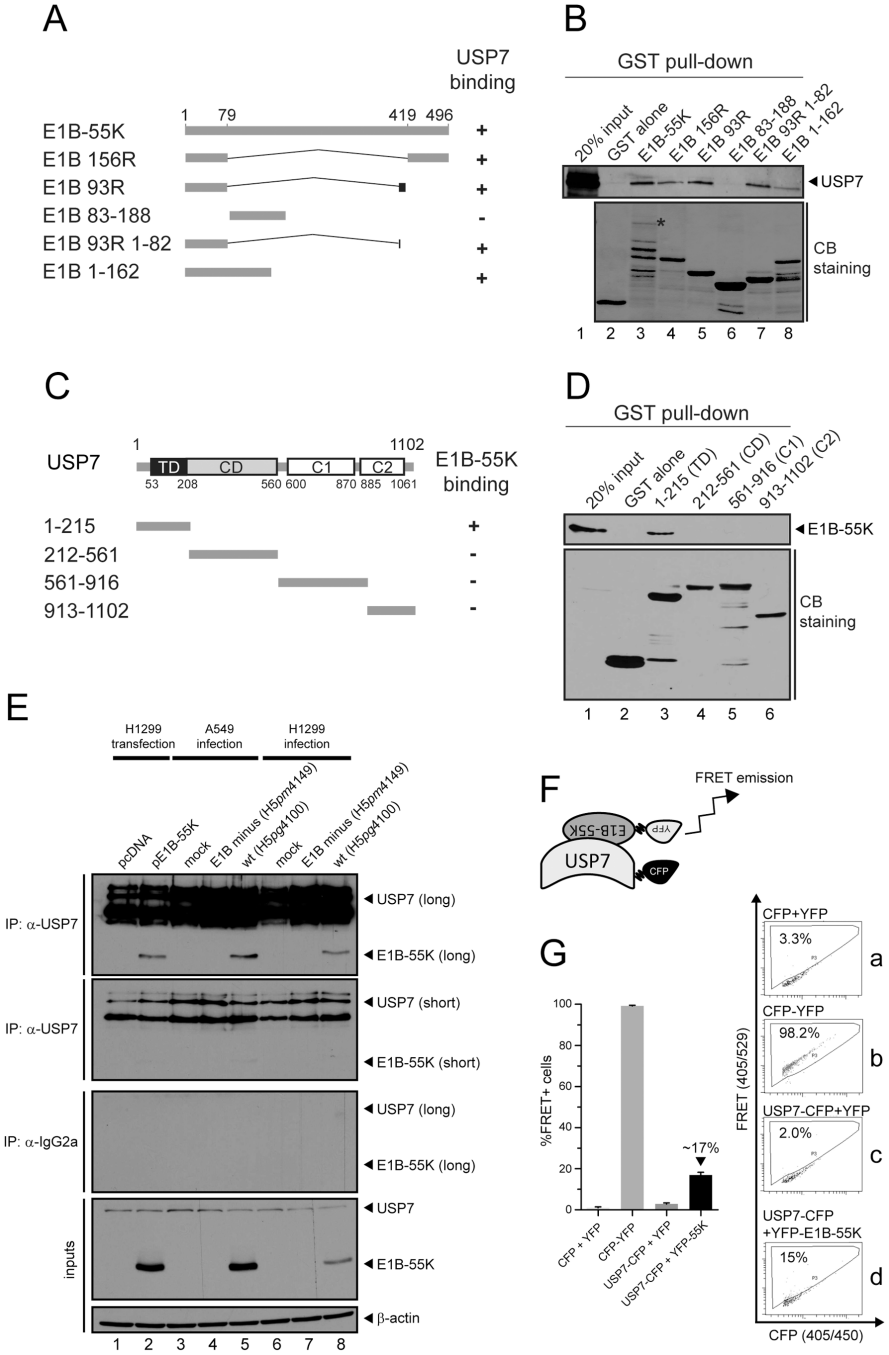
USP7 interacts directly with E1B-55K. (A) Schematic representation of E1B-55K splice-variants as indicated. Numbers refer to amino acid residues of HAdV5 E1B-55K protein. A summary of each construct's ability to bind USP7 is shown (+/−). (B) Similar amounts of bacterially expressed GST fusion products immobilized to Glutathione-sepharose beads were incubated with extracts of H1299 cells. Precipitated proteins were subjected to immunoblotting (IB) to detect USP7 (lanes 1 and 3–8). GST fusion proteins were visualized by Coomassie brilliant blue (CB) staining. The asterisk indicates the position of the full-length GST fused E1B-55K. (C) Segments corresponding to the four structural domains of USP7 are indicated and a summary of their ability to bind to E1B-55K. (D) GST fused USP7 segments were incubated with HAdV5-infected H1299 cell extracts prepared at 48 h p.i. Input and precipitated proteins were subjected to IB to detect E1B-55K. Visualization of GST fusions was performed as in (B). (E) H1299 cells were mock-transfected with vector pcDNA3 and vector encoding E1B-55K and harvested 30–40 hours post transfection (h p.t.). Moreover, A549 and H1299 cells either mock-infected or infected (MOI = 20 FFU/cell) with indicated viruses were harvested 24 hours post infection (h p.i.) and subjected to coimmunoprecipitation, together with transfected H1299 cell lysates, with the USP7 antibody 6E6 and control antibody IgG2a. Proteins were subjected to IB with antibodies against E1B-55K, USP7 and β-actin. (F) Cartoon showing FRET emission after interaction of YFP- and CFP-tagged proteins. (G) H1299 cells were transfected in indicated combinations with 1–8 µg of pEYFP/pECFP, pECFP-YFP (fusion), USP7-CFP or YFP-E1B-55K and PEI (1 µg/µl) in a ratio of 1∶4 or 1∶5, harvested 48 h p.t. and analyzed via FACS. S.e.m. from a minimum of six independent experiments is summarized. Representative primary FACS-plots are presented on the right (a–d).

Next, we wanted to verify the results from the yeast two-hybrid screen *in vitro*. Therefore, as summarized in Figure 1A, we generated GST fusion proteins including full-length, and a series of truncated or alternative splicing variants of E1B-55K protein and evaluated their interaction with cellular USP7 by GST pull-down experiments. GST purification of intact full-length E1B-55K protein was inefficient since it proved unstable when expressed in bacteria (Figure 1B, lower panel, lane 3). However, the combination of full-length protein and its bacterial degradation products could precipitate USP7 (Figure 1B, upper panel, lane 3). Besides the full-length protein, all the fusion products containing the first 79 residues of E1B-55K precipitated USP7 regardless of their C-terminal extensions (Figure 1B, upper panel, lanes 4, 5, 7 and 8). Taken together, these data demonstrate that the N-terminal 79 amino acid region of E1B-55K is necessary and sufficient for binding to USP7 *in vitro*.

Previous investigations had revealed that the USP7 protein can be roughly divided into four domains [34]–[36]: the N-terminal TRAF-like domain (TD; residues 53–208), central catalytic domain (CD; residues 208–560), and two C-terminal structural domains (C1 and C2; residues 600–870 and 885–1061, respectively) (Figure 1C). To identify which of these domains interacts with E1B-55K, we generated GST fusions corresponding to these regions and carried out GST pull-down experiments in wt (H5*pg*4100)-infected H1299 cell extracts (Figure 1D). Neither the central CD, nor the C-terminal domains C1 and C2 interacted with E1B-55K; however, the N-terminal segment of USP7 (residues 1–215) was found to strongly and specifically precipitate E1B-55K (Figure 1D, lane 3).

To further investigate the USP7 interaction, endogenous USP7 was immunoprecipitated from p53-negative H1299 cells transfected with a plasmid encoding wt pE1B-55K and stained for coprecipitated E1B-55K (Figure 1E, lanes 1 and 2). Additionally, H1299 (p53-negative) and A549 (p53-positive) cells were mock-infected and infected with E1B minus (H5*pm*4149) or wt (H5*pg*4100) virus. Subsequent USP7 immunoprecipitation experiments confirmed USP7-E1B-55K interactions in both cell lines (Figure 1B, lanes 5 and 8) whereas control immunoprecipitation experiments with an unspecific IgG2a antibody proved to be negative for E1B-55K as well as USP7 precipitation (Figure 1B).

To verify this interaction in living cells, flow cytometry-based FRET (Foerster's Resonance Energy Transfer) analyses were employed as described by Banning and colleagues [37], with USP7-CFP serving as a donor chromophore and YFP-E1B-55K as an acceptor chromophore (scheme, Figure 1F). When both proteins interact, excitation of the CFP chromophore results in secondary excitation of the YFP chromophore leading to FRET signal emission displayed in the corresponding FRET gate (Figure 1G, panel b or d). False-positive signals are excluded by using different controls, as indicated, together with an appropriate gating strategy (Figure 1G, panel a and c). This assay revealed ca. 17% FRET-positive cells (FRET+; Figure 1G, graph + panel d), indicating that USP7 and E1B-55K also interact in living cells. Although FRET+ cell levels were relatively low compared to the positive control (fusion of CFP and YFP resulting in “constant” FRET emission; CFP-YFP), possibly explained by CFP/YFP-tag interference and/or competition between endogenous and exogenous USP7, nevertheless, FRET+ cells scored significantly more than the negative controls (CFP cotransfected with YFP; compare Figure 1G, panels a and d).

Taken together our results establish E1B-55K as a new specific interaction partner of USP7 which could also be confirmed in living cells.

### USP7 is relocalized during infection into viral replication centers

**Figure 2:**
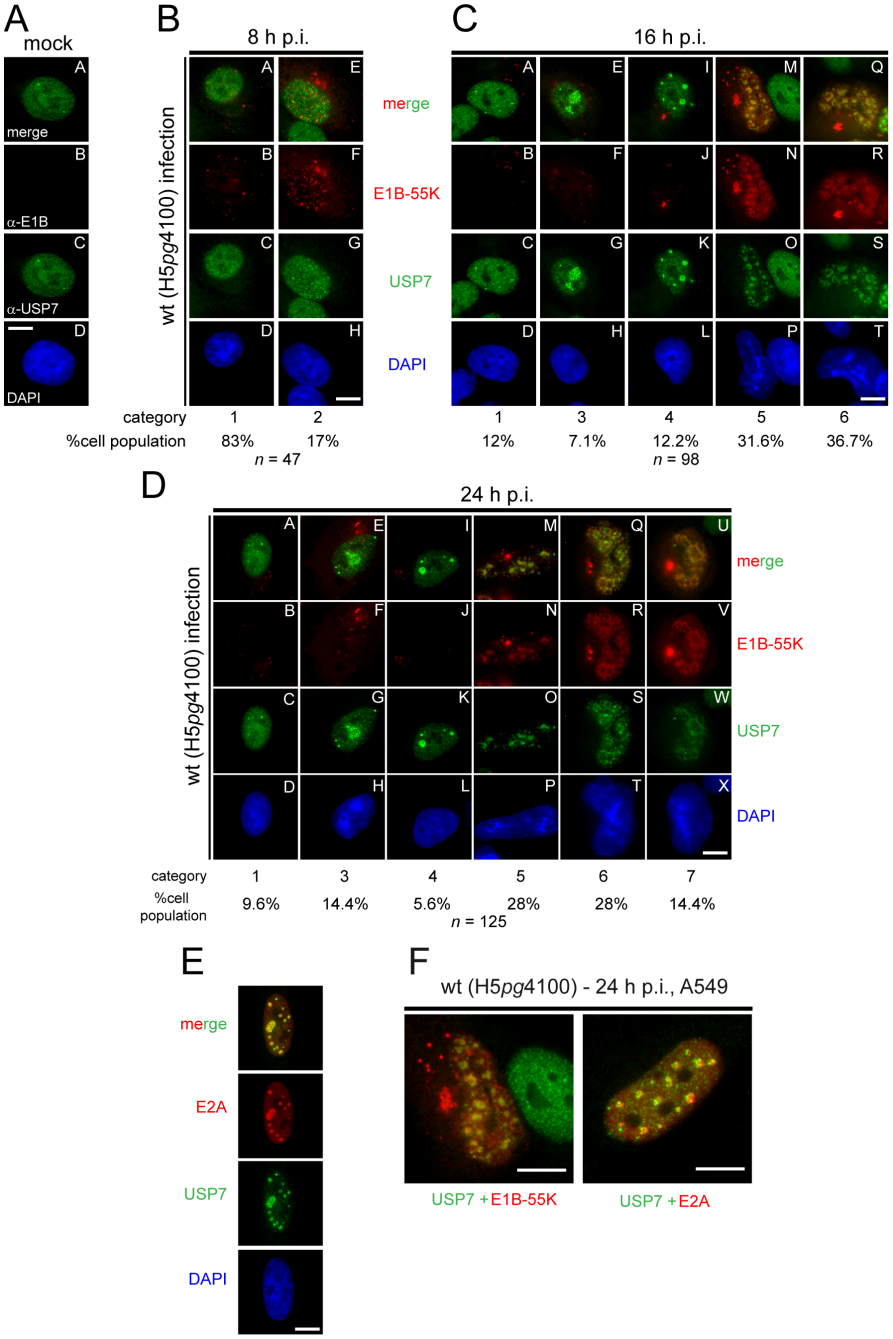
Subcellular localization of E1B-55K, E2A and USP7 in virus infected cells. (A) Mock-infected A549 cells were analyzed by *in situ* immunofluorescence staining for E1B-55K (2A6, red), USP7 (3D8, green), and DNA content (DAPI, blue). (B–D) A549 cells infected with wt virus (H5*pg*4100) (MOI = 20 FFU/cell) were analyzed by *in situ* immunofluorescence staining for E1B-55K (2A6), USP7 (3D8), and DNA content (DAPI) at indicated time points. Represented are the major phenotypes observed in A549 cells, categorized as 1–7, with the percentage of the population in each category indicated. (E) Same procedure as in (B–D). However, cells (16 h p.i.) were analyzed by *in situ* immunofluorescence staining for E2A (B6-8), USP7 (3D8), and DNA content (DAPI, blue). (F) Wt-infected (H5*pg*4100) A549 cells 24 h p.i. (MOI = 20, FFU/cell) costained for USP7 (3D8, green) and E1B-55K (2A6, red) (left) compared to USP7 (3D8, green) and E2A (B6-8, red) (right). White bars represent 10 µm length.

To determine if adenoviral infection affects USP7 subcellular localization in a time-dependent manner, we performed extensive time course immunofluorescence studies. A549 cells were mock-infected or infected at an MOI of 20 FFU per cell with wt HAdV5 virus (H5*pg*4100) and then methanol-fixed at indicated hours post infection (h p.i.; Figure 2A to D). USP7 and E1B-55K (E1B) were detected with specific monoclonal antibodies, and visualized using double-label immunofluororescence microscopy. In uninfected cells (mock), USP7 localized diffusely in the nucleus with a few prominent dot-like structures (Figure 2A). However, upon wt adenoviral infection (H5*pg*4100), USP7 localization changed dramatically. Several different relocalization patterns of USP7 were observed, which were categorized for each investigated time point with respect to E1B-positive cells (percentages of each category are denoted in Figure 2B to D). In general, USP7 increasingly accumulated into dense, ring-like structures over time during adenovirus infection. Interestingly, as infection proceeds USP7 colocalization with E1B-55K increasingly correlates with these ring-like structures (merge). This is especially displayed in cells with USP7 relocalization of categories 5 and 6.

However, the initial step of USP7 redistribution is probably independent of E1B-55K, since USP7 relocalization could be observed before detection of E1B-55K, and in the absence of colocalization (Figure 2C, panels E–H and I–L) and during infection with a virus lacking all E1B functions (Figure S2, panels D–F). Nevertheless, USP7 redistribution in the nucleus forming ring-like structures as seen in categories 5, 6 and 7 was observed in the majority of patterns analyzed up to 24 hours post infection (Figure 2C, panels M–T; 2D panels M–X).

Strikingly, the relocalization pattern of USP7 during wt adenoviral infection (H5*pg*4100) strongly resembled staining patterns of the adenoviral E2A protein (also called DBP, Figure 2E and F) [38]–[41]. E2A is a single-stranded DNA (ssDNA) binding protein involved in adenoviral genome replication and can be found not only colocalized with sites of viral ssDNA, but also surrounding sphere-shaped sites of double-stranded DNA, and is thus a marker for sites of both transcription and replication [42], [43].

To test whether USP7 is relocalized to sites of viral DNA replication and transcription, *in situ* costainings of E2A and USP7 were prepared to detect significant colocalization of both proteins in a time-dependent manner (from 8–48 h p.i. data not shown). In a similar approach as above, A549 cells were infected at an MOI of 20 FFU/cell and methanol-fixed at different time points. Upon analyzing the staining patterns of USP7 in E2A-positive cells, it was evident that nearly all the cells displayed USP7 staining patterns strongly correlating with the E2A-stained structures which is exemplified in Figure 2E or 2F (16 h p.i. or 24 h p.i.).

Altogether, these observations demonstrate that time-dependent USP7 relocalization is strictly related to the formation of viral replication centers, where interaction with E1B-55K probably occurs. Moreover, this points to a functional exploitation of the cellular DUB like it has been shown for a number of other cellular proteins relocalized to adenoviral replication centers (e.g. BLM, RPA32, Mre11, ATR, ATRIP, E1B-AP5 [44]–[46]).

### Establishing USP7 knockdown and inhibition conditions

To examine the role of USP7 in adenoviral infection, we analyzed the effects of reducing USP7 steady-state levels and inhibiting USP7 on E1B-55K protein levels and adenoviral replication.

**Figure 3:**
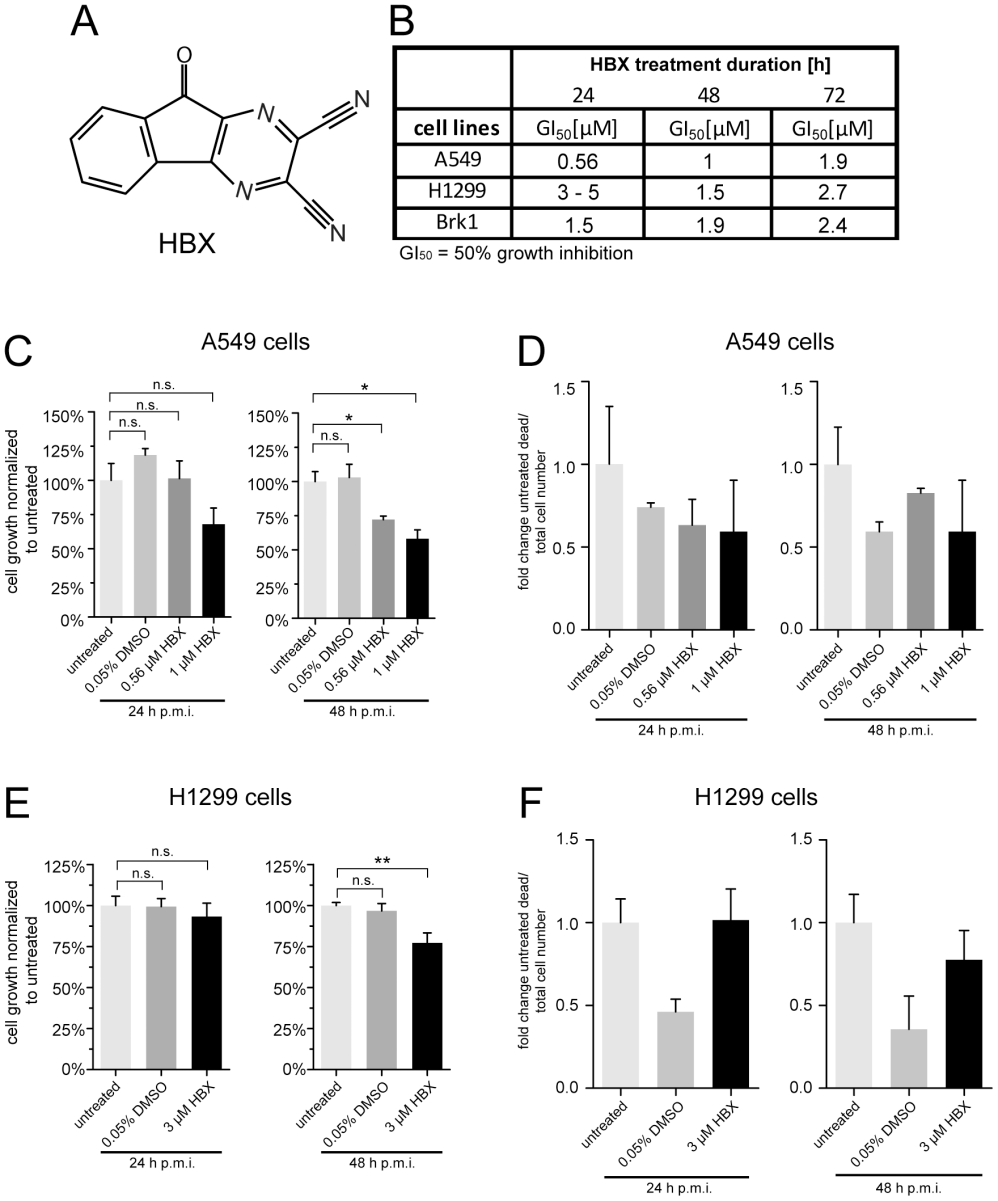
Summarized GI_50_ values and growth behavior of cells after HBX treatment. (A) Chemical structure of the USP7 inhibitor HBX. (B) Several growth inhibitory 50 (GI_50_) values were determined as described in Materials and Methods and summarized. (C+E) Analysis of the growth behavior of A549 and H1299 cells upon HBX treatment. 1.5×10^6^ cells were seeded 15–20 h before HBX treatment. Cells were treated with HBX or DMSO for a total of 15 h and harvested 24 or 48 hours post mock infection (h p.m.i.) and counted. S.e.m. of at least 3 independent experiments is shown. P-values of unpaired, two-tailed t-tests (*P<0.05, **P<0.01, n.s. = not significant). (D+F) The number of dead cells (trypan blue positive) was divided by the total cell number and then normalized to the number of untreated cells. Trypan blue positive cells were counted after using same experimental conditions as in (C+E).

We generated USP7 knockdown and corresponding control cell lines (using H1299 and A549 as parental cell lines) and synthesized a small-molecule compound [47] (here called “HBX”; Figure 3A) which was previously shown to inhibit USP7 [48]. Moreover, we utilized a second USP7 inhibitor (HBX41108), which is a derivative of HBX, to support specificity of our assays [47], [48].

To assess optimal conditions for the inhibitor assays, growth behavior and viability of the cells were tested under mock infection conditions plus inhibitor treatment. In a first attempt to characterize HBX, MTS-based proliferation assays were carried out on cell lines used in our experiments. As shown in Figure S3, sigmoidal dose response curves were generated for three inhibitor treatment durations (24, 48 and 72 h) with several dilution rows performed at least in triplicate. The summarized GI_50_ values are represented in Figure 3B and reveal that HBX administration in the micromolar range results in growth inhibition.

Previous reports demonstrated that loss of USP7 through knockout leads to decreased proliferation of the respective cell line [49]. Therefore, since USP7 plays a critical role in cell proliferation, it was necessary to determine inhibitor treatment conditions that did not significantly inhibit cell growth. Otherwise it would be difficult to distinguish between cell growth defects or specific compound-mediated effects negatively influencing virus yield. First, time-of-addition experiments after mock infection were performed to determine tolerable inhibitor concentrations leading to insignificant cell growth inhibition. In effect, it turned out that 15 hours of inhibitor treatment prior to cell harvest worked best for all investigated cell lines. For example, A549 cells exhibited no statistically significant decrease in cell number compared to untreated cells 24 hours post mock infection (h p.m.i.) at both HBX concentrations (Figure 3C). However, a significant reduction in cell numbers was observed 48 h p.m.i. after HBX application at both concentrations (∼25% reduction). Nevertheless, trypan blue exclusion to determine the number of viable cells displayed no significant cytotoxic effect on A549 cells either 24 or 48 h p.m.i., meaning that cell cytotoxic effects could be excluded in subsequent experiments (Figure 3D). Similarly, H1299 cells experienced no cell growth defect after HBX treatment 24 h p.m.i., but underwent ca. 25% reduction 48 h p.m.i. (Figure 3E). However, again the number of dead cells did not increase after HBX incubation compared to untreated cells (Figure 3F).

**Figure 4:**
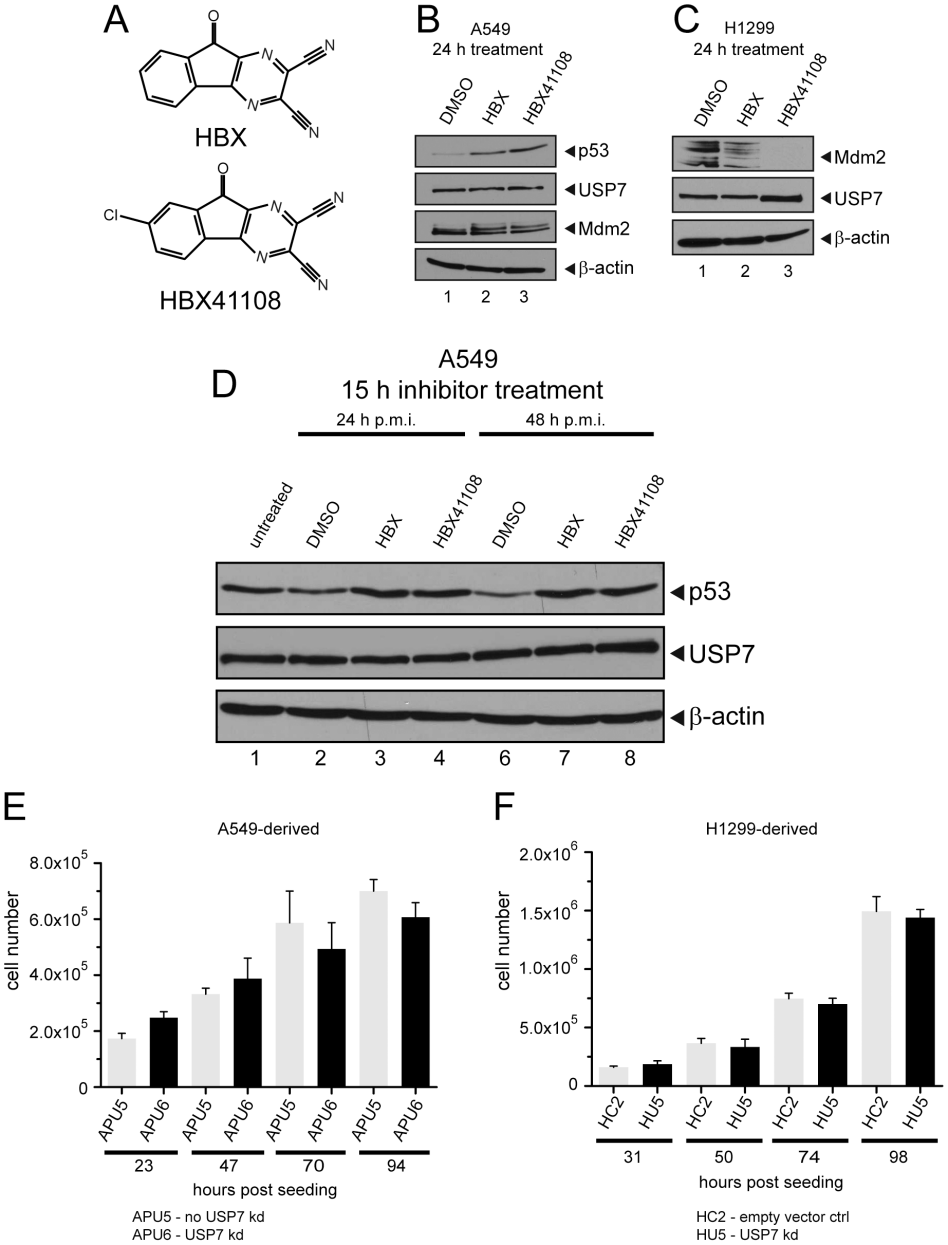
USP7 inhibitor specificity and growth behavior of knockdown cell lines. (A) Chemical structure of the USP7 inhibitor HBX and its derivative HBX41108. (B) A549 cells were treated with DMSO, HBX (0.56 µM end concentration) and HBX41108 (0.56 µM end concentration) for 24 hours. Proteins were subjected to immunoblotting (IB) with antibodies against p53, USP7, Mdm2 and β-actin. (C) H1299 cells were treated with DMSO, HBX (3 µM end concentration) and HBX41108 (3 µM end concentration) for 24 hours. Proteins were subjected to IB with antibodies against USP7, Mdm2 and β-actin. (D) A549 cells were harvested 24 or 48 hours post mock infection (h p.m.i.). 15 hours before cell harvest, cells were treated with DMSO, HBX (0.56 µM end concentration) or HBX41108 (0.56 µM end concentration). Untreated cells were harvested 48 h p.m.i. (E+F) APU5 and APU6 or HC2 and HU5 cells were harvested at indicated hours post seeding and the number of viable cells was determined. Results represent the mean values of at least three independent experiments. APU5 and APU6 were transfected with the same plasmid construct for USP7 knockdown but APU5 displays no knockdown of USP7 (no USP7 kd).

Previous reports have shown that inhibitors of USP7 such as HBX41108 (Figure 4A) induce, among others, p53 protein accumulation and a decrease in Mdm2 protein levels [48], [50]–[52]. This is because, upon USP7 inhibition, Mdm2 deubiquitination/stabilization is heavily decreased and auto-ubiquitination of Mdm2 increased with subsequent lower p53 turnover and p53 accumulation [35].

**Figure 5:**
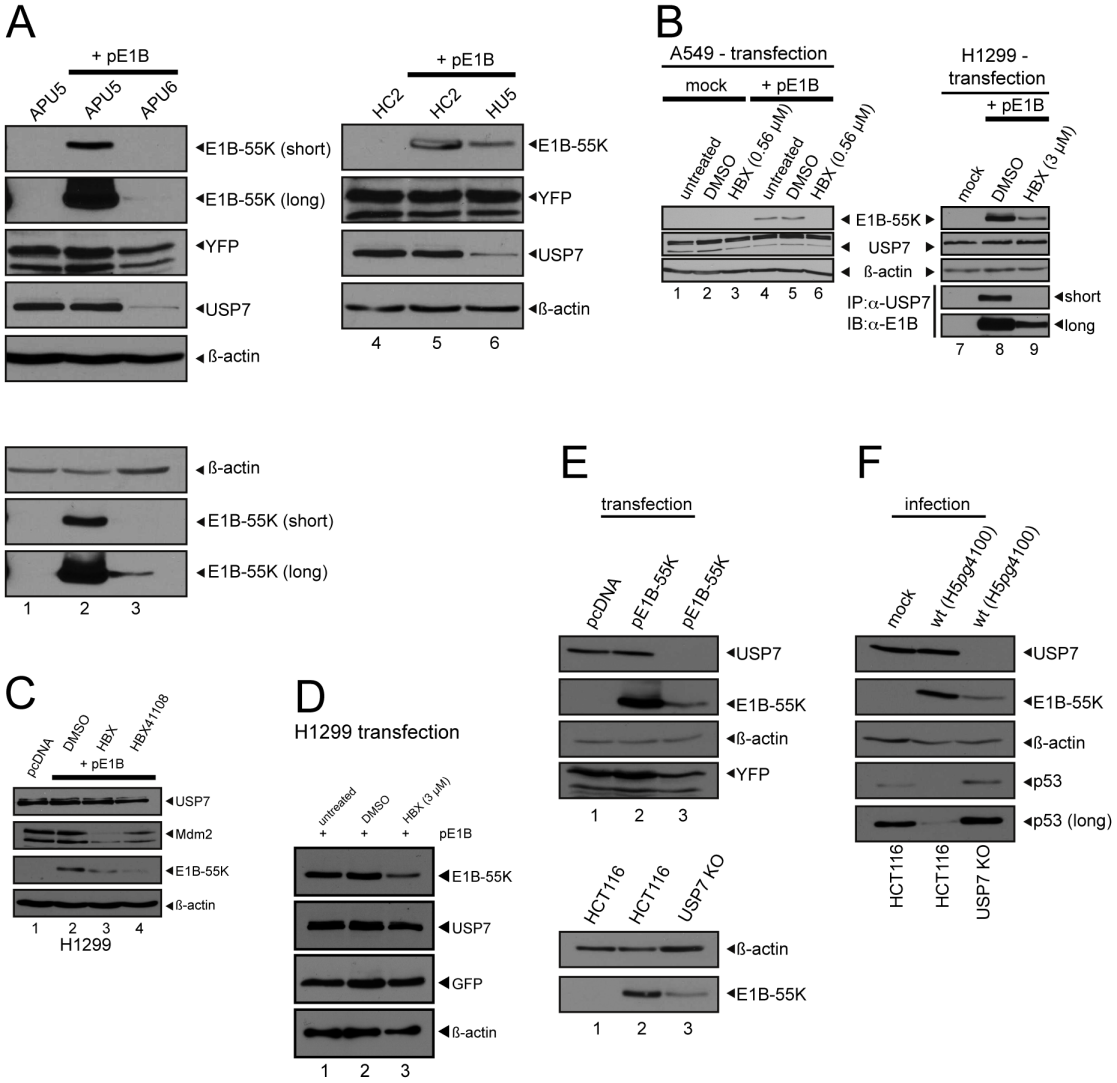
USP7 knockdown, knockout or inhibition reduces E1B-55K levels. (A) Equal numbers of the indicated cells were seeded, and transfected with pcDNA3 (3 µg) or plasmid vector containing E1B-55K (3 µg, pE1B-55K) and pEYFP (1 µg HC2/HU5 or 2 µg APU5/APU6). 24–30 hours post transfection (29–40 h p.t. APU5/APU6), cells were harvested, lysed and immunoblotted. Antibodies detecting E1B-55K (2A6), USP7 (3D8), GFP/YFP (sc8334) and β-actin (AC-15) were used. (B) A549 or H1299 cells were transfected with 2 µg plasmid vector pcDNA3 (mock) or with 2 µg pE1B-55K (wt E1B-55K; pE1B). Cells were treated with DMSO, HBX (0.56 µM/3 µM end concentration, added 6 h p.t.) or left untreated. Cells were harvested 30 h p.t., lysed and immunoblotted. Antibodies detecting E1B-55K (2A6), USP7 (3D8), and β-actin (AC-15) were used. Additionally, H1299 cells were subjected to coimmunoprecipitation with USP7 antibody 3D8 similarly performed as in Figure 1E. (C) H1299 cells were transfected with 2 µg plasmid vector pcDNA3 (mock) or with 2 µg pE1B-55K (wt E1B-55K; pE1B). Cells were treated with DMSO, HBX and HBX41108 (3 µM end concentration, added 9 h p.t.) or left untreated. Cells were harvested 24 h p.t., lysed and immunoblotted. Antibodies detecting E1B-55K (2A6), USP7 (3D8), Mdm2 (AF1244) and β-actin (AC-15) were used. (D) H1299 cells were transfected with 3 µg pE1B-55K (wt E1B-55K; pE1B) and 1 µg of a plasmid vector containing GFP. Cells were treated with DMSO and HBX (3 µM end concentration, added 9 h p.t.) or left untreated. 24 h p.t. cells were lysed and immunoblotted. Antibodies detecting E1B-55K (2A6), USP7 (3D8), GFP (sc8334) and β-actin (AC-15) were used. (E) Equal numbers of HCT116 and HCT116 USP7 KO cells were seeded, transfected with 4 µg pcDNA, 4 µg pE1B-55K and 2 µg pEYFP. 25–35 h p.t. cells were lysed and immunoblotted. Same antibodies as in (A) were used. In a second Western blot, double amounts of USP7 KO protein lysate were assayed (new staining). (F) Equal numbers of HCT116 and HCT116 USP7 KO cells were seeded, infected with wt adenovirus (H5*pg*4100; MOI 50 FFU/cell) and analyzed 24 h p.i. Antibodies detecting E1B-55K (2A6), USP7 (3D8), p53 (DO-1) and β-actin (AC-15) were used.

In order to assess whether HBX exerts similar effects on Mdm2 and p53, the two cell lines mainly used in this study were treated with HBX and HBX41108 for 24 or 15 hours (assay conditions). As expected, both compounds induce an increase in the steady-state protein levels of p53 in A549 cells (Figure 4B, lanes 2 and 3; Figure 4D, lanes 3, 4, 7 and 8). However, a decrease of Mdm2 protein levels could not be detected in this cell line either owing to deregulation of the USP7-p53-Mdm2 pathway or low/too short inhibitor treatment duration. Nevertheless, a decrease in Mdm2 protein levels could be detected in H1299 cells (Figure 4C, lanes 2 and 3; Figure 5C, lanes 3 and 4).

USP7 knockdown (kd) cell lines were generated as an additional tool for this study. The A549-derived USP7 knockdown cell line APU6 (Figure 5A, lane 3) displayed slower growth rates than the parental cell line (data not shown). However, another cell line also transfected with the shRNA plasmid construct against USP7 and derived from A549 cells, APU5, with slow growth comparable to APU6 cells presents normal USP7 levels (Figure 5A, lane 1). To exclude the possible influence of slower growth, this APU5 cell line was used as a control (Figure 4E). Similarly, the USP7 knockdown cell line HU5 derived from H1299 cells (Figure 5A, lane 6) possessed almost identical growth rates compared to the control cell line HC2 (Figure 4F) and the H1299 parental cell line (data not shown).

In summary, it was possible to establish two USP7 knockdown cell lines with corresponding control cells, and find suitable conditions for HBX treatment in infection experiments where cell growth and viability were not significantly affected. Nonetheless, cell growth defects after USP7 inhibition were observed at later stages of mock infection, which corresponds to previously described effects of USP7 on cell proliferation. Therefore, these effects were expected and taken into consideration in subsequent experiments (by normalizing the virus yield per cell and subsequently normalizing yield without inhibitor treatment). Additionally, as expected, both compounds HBX and HBX41108 were able to induce an increase in p53 protein levels but also a decrease in Mdm2 protein levels indicating specific inhibitory effects exerted upon USP7.

### USP7 knockdown/knockout or inhibition reduces E1B-55K steady-state levels

When we transfected E1B-55K expression constructs along with an expression construct for EYFP (YFP) into the USP7 knockdown and corresponding control cell lines, we detected severely reduced E1B-55K steady-state levels in the USP7 knockdown background (HU5) without affecting expression levels of the control plasmid encoding YFP (Figure 5A, lane 6). However, a slight reduction of YFP was detected in APU6 cells (Figure 5A, lane 3). Therefore same samples were reanalyzed by Western blot (Figure 5A, lower panel) with double amounts of APU6 (lane 3) in comparison to APU5 lysates (lanes 1 and 2). Here, in APU6 USP7 knockdown cells (lane 3) E1B-55K protein levels still displayed strong reduction in comparison to APU5 cells with normal USP7 protein levels (lanes 1 and 2). To support the knockdown data, we treated A549 and H1299 parental cell lines with the USP7 inhibitors HBX (Figure 5B; Figure 5C; Figure 5D) or HBX41108 (Figure 5C, lane 4). Neither DMSO nor HBX/HBX41108 affected steady-state protein levels of USP7, but in HBX/HBX41108-treated cells protein levels of transfected E1B-55K were severely reduced, comparable to the knockdown experiments (Figure 5B, lanes 6 and 9). Moreover, coimmunoprecipitation of E1B-55K by USP7 was reduced after treatment with HBX (Figure 5B, lane 9) and HBX did not display reduction of cotransfected GFP (Figure 5D).

Additionally, transfecting an E1B-55K expression construct into HCT116 USP7 knockout cells (USP7 KO) also resulted in strong reduction of E1B-55K protein levels (Figure 5E, upper panel). An identical result was obtained when HCT116 lysates (Figure 5E, lower panel, lanes 1 and 2) were compared to double amounts of USP7 KO lysates (Figure 5E, lower panel, lane 3) by immunoblotting. The same effect was obtained after infection of the respective cell lines (Figure 5F). Corresponding p53 staining showed significantly increased p53-levels despite infection with wt (H5*pg*4100) virus at an MOI of 50 FFU per cell indicating insufficient adenoviral E3 ligase activity due to low E1B-55K protein levels (Figure 5F).

The stabilizing effect of USP7 upon E1B-55K was further supported by cycloheximide chase assays demonstrating a reduced half-life of E1B-55K in the USP7 knockdown background (Figure S4A) and after HBX or HBX41108 treatment (Figure S4B and C).

Taken together, USP7 knockdown/knockout or inhibition led to greatly reduced E1B-55K protein levels, indicating a stabilizing role of USP7 for E1B-55K.

### Adenovirus replication is reduced in USP7-depleted cells and when USP7 is inhibited after infection

Herpesviruses, like HSV-1 and KSHV rely on the functions of USP7 to efficiently promote virus growth or genome replication [29], [53]. In contrast, the role of cellular DUBs in adenovirus replication has not been investigated.

**Figure 6:**
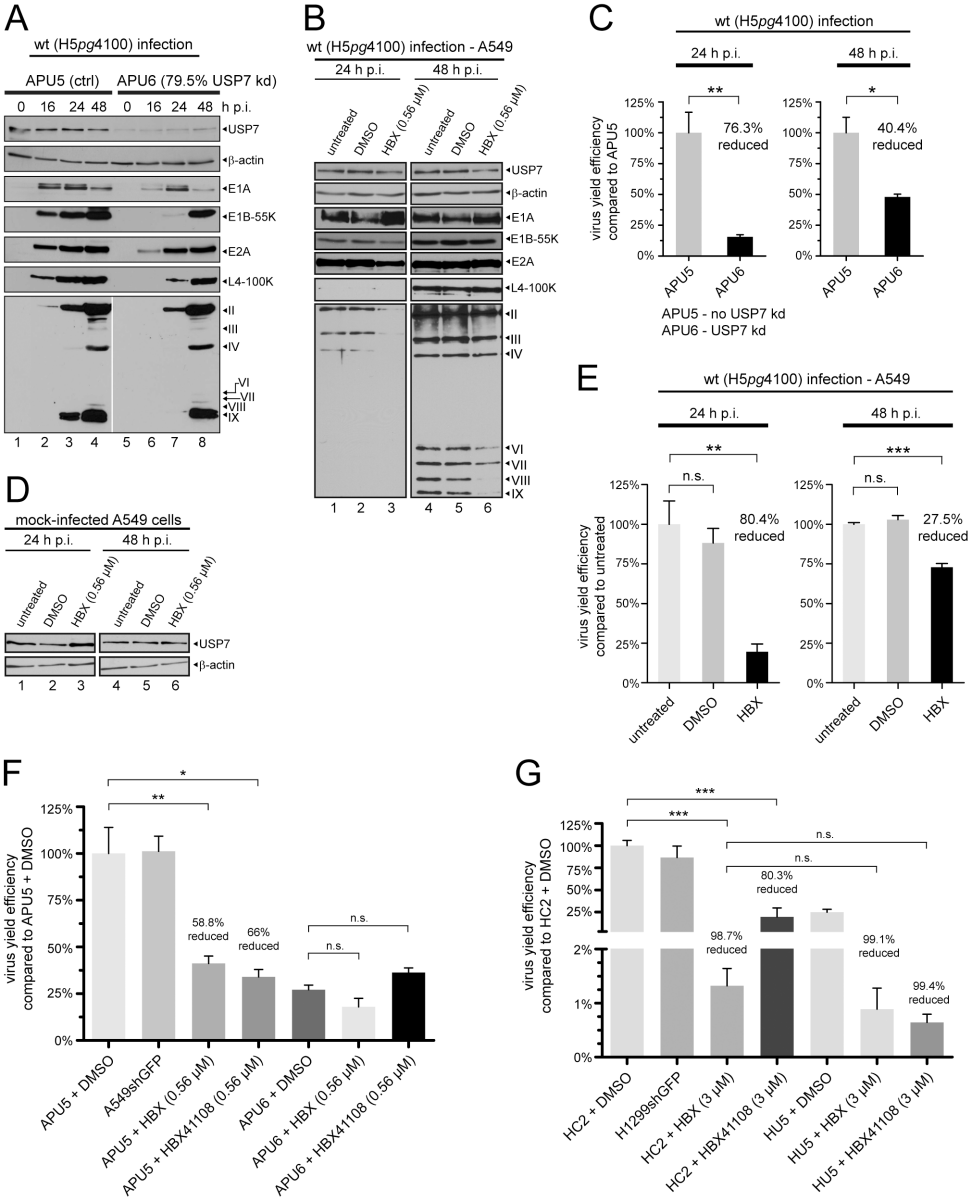
USP7 knockdown or inhibition has negative effects on adenoviral proteins and virus growth. (A) APU5 and APU6 cells were infected with wt virus (MOI 20 FFU/cell). Cell extracts were prepared at indicated h p.i. and subjected to IB to detect USP7, β-actin, E1A, E1B-55K, E2A, L4-100K and viral late proteins (Roman numerals; originating from one blot, leaving out empty marker lane). ImageJ USP7 band intensity quantification was performed. (B) A549 cells were infected as in (A), but harvested at 24 and 48 h p.i. and analyzed to detected indicated proteins. 15 h before cells were harvested, DMSO or HBX was added to the cells as indicated. (C) APU6 and APU5 cells were infected with wt virus (MOI 20 FFU/cell). Infected cells were harvested at 24 and 48 h p.i. and infectious virus particles produced were determined by quantitative E2A staining on HEK293 cells. At least four independent experiments were performed. Error bars indicate the standard error of the mean of viral particles per cell which were normalized to APU5 cells. P-values of unpaired, two-tailed t-tests (*P<0.05, **P<0.01). (D) A549 cells were treated as in (B) except without adding virus particles. (E) Same procedure as in (B), but at indicated time points the virus yield was determined as in (C). At least three independent experiments were performed. Bars indicate the standard error of the mean values. Virus yield efficiency is represented as a percentage of untreated wt infected A549 cells. P-values of unpaired, two-tailed t-tests (*P<0.05, **P<0.01, ***P<0.001). (F+G) Indicated cell lines were infected and virus yield determined 24 h p.i. as in (E+S5E).

In a first step to evaluate the influence of USP7 on adenovirus infection, the generated USP7 kd cells APU6 and HU5 (79.5% and 86.6% knockdown efficiency respectively; Figure 6A and S5A) were infected with wt virus (H5*pg*4100), and the synthesis of early and late viral proteins, as well as the production of progeny virions were compared to those of the control cell lines at different time points (Figure 6 and S5).

First, the effect of USP7 depletion on the synthesis of early viral proteins E1A, E1B-55K and E2A was assayed by immunoblotting. Surprisingly, being the first gene products expressed, E1A proteins showed a defect in accumulating protein levels in APU6 and HU5 cells compared to the USP7+ counterparts. Similarly, E2A levels were also detected to be slightly lower in these cells than in the control USP7+ APU5 and HC2 cells. Consequently, when the expression pattern of E1B-55K was investigated, a significant defect was observed not only in the expression time, but also in the amounts of this protein (Figure 6A and S5A).

The expression of late structural proteins was also investigated in the knockdown cells. As expected, the observed inefficient synthesis of the early viral proteins resulted in delayed accumulation of late structural proteins in both APU6 and HU5 cells compared to the USP7+ counterparts during wt infection (H5*pg*4100). Late structural protein synthesis was either delayed in USP7 kd lines (e.g. pIII in HU5, or minor capsid proteins in APU6), or these proteins did not accumulate to the parental cell line levels (e.g. pVI in HU5 or pII in both knockdown lines) (Figure 6A and S5A).

Moreover, in order to investigate whether USP7 inhibition leads to effects similar to USP7 knockdown, infected H1299 and A549 cells were subsequently treated with inhibitor HBX. USP7 protein steady-state levels were not affected after inhibitor treatment, either in infected or mock-infected cells in both cell lines (Figure 6B and D; Figure S5C and D). Similar to the knockdown experiments, a reduction of E1B-55K and structural capsid proteins could be detected after HBX treatment in both cell lines (Figure 6B and S5C, each lane 3). Less E1B-55K was further confirmed by quantifying E1B-positive cells after HBX treatment of infected cells using immunofluorescence microscopy (Figure S6). However, decreased E1B-55K could only be observed 24 h p.i., but not 48 h p.i., consistent with the immunofluorescence quantification data (compare Figure 6B and S5C with Figure S6B and D). This may suggest that functional inhibition of USP7 cannot overcome the likely high transcription-translation activity at this stage of infection (at least for the early protein E1B-55K). Interestingly, E1A levels seemed to increase whereas E2A levels only show a slight decrease in A549 cells, and L4-100K protein levels displayed a modest decrease after HBX incubation (Figure 6B and S5C, each lane 3). It is probable that differences between both approaches (knockdown vs. inhibition) may reflect variable efficiencies of functional inhibition.

However, overall, knockdown or inhibition of USP7 led to reduced steady-state protein levels of various adenoviral proteins.

Virus yield experiments performed in both USP7 kd and their respective control cell lines, demonstrated 76.3% or 72.5% reduced viral progeny numbers 24 h p.i. in APU6 or HU5 (Figure 6C and S5B). At 48 h p.i. the virus yield was still reduced by 40.4% (APU6) or 26% (HU5), implying that USP7 is biologically significant for efficient adenovirus infection, even at the late stage of infection.

**Figure 7:**
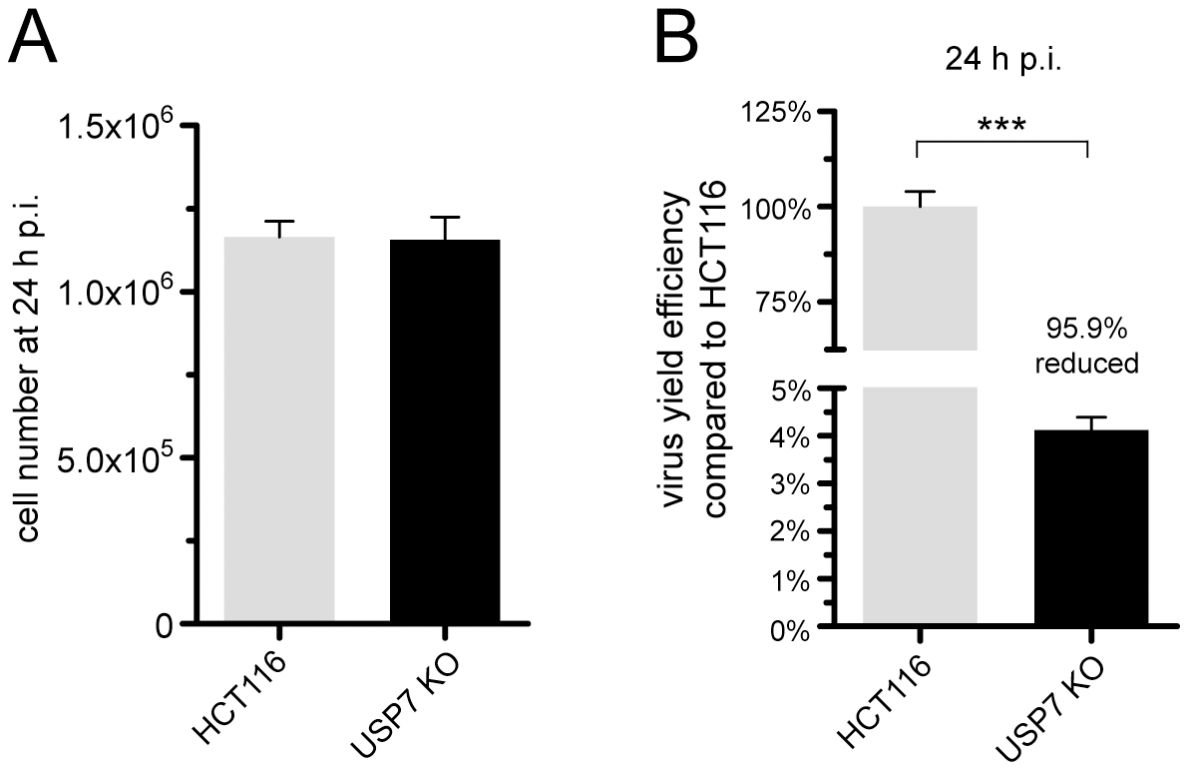
USP7 knockout has severe effects for adenoviral progeny production. (A+B) HCT116 and HCT116 USP7 double-knockout cells (USP7 −/− [KO]) were infected with wt virus (H5*pg*4100; MOI 50 FFU/cell). Cells were harvested 24 h p.i. and for each sample cell number was determined. Virus extraction was performed as in Figure 6C.

More importantly, USP7 KO cells, devoid of any USP7 function, were infected with wt virus (H5*pg*4100) along with the respective control cell line HCT116. The USP7 KO cells were kept in 15%FBS containing medium to compensate for the growth defect this cell line exhibits in comparison to HCT116 cells [49], [54]. Nearly identical numbers of wt-infected HCT116 and USP7 KO cells were harvested at 24 h p. i. (Figure 7A) and virus yield was determined (Figure 7B). As expected, the number of infectious virus progeny particles was severely diminished up to 95.9% (Figure 7B) even though a relatively high MOI of 50 FFU per cell was used. These results strongly support the findings that USP7 is required for efficient adenovirus infection.

As with the knockdown experiments or in the USP7 KO background, inhibitor treatment (15 h before assaying) strongly impaired virus growth in A549 and H1299 cells 24 h p.i. (A549 = 80.4% and H1299 = 91.4%, Figure 6E and S5E). Even at a later time point (start 33 h p.i. with harvest 48 h p.i.) structural capsid proteins (Figure 6B and S5C, each lane 6) and virus progeny numbers (Figure 6E and S5E) were significantly reduced (27.5% A549 and 44.1% H1299). This clearly supports the notion that USP7 may exert its effects not only during early, but also at late times of infection. Moreover, the degree of virus growth inhibition was comparable to that after USP7 knockdown (compare Figure 6E and C). To exclude off-target effects and to demonstrate specificity towards USP7 virus yield was determined in cell lines expressing an shRNA against GFP (A549shGFP and H1299shGFP) and compared to the control cell lines APU5 and HC2 (Figure 6F and G). No significant reduction in virus progeny production was observed in A549shGFP and H1299shGFP cells, respectively (Figure 6F and G). Furthermore, another USP7 inhibitor, HBX41108, demonstrated similar efficacy in reducing adenoviral progeny numbers as HBX (Figure 6F, HBX 58.8% reduction, HBX41108 66% reduction; Figure 6G, HBX 98.7% reduction, HBX41108 80.3% reduction).

Next, our knockdown cell lines were treated with both USP7 inhibitors. In APU6 cells neither HBX nor HBX41108 could further significantly reduce virus yield (Figure 6F). This indicates that the effects observed in our hands are specific to USP7 inhibition. Interestingly, in HU5 cells further reduction of progeny virus numbers could be achieved, but this reduction is comparable to that after HBX treatment in the HC2 control cell line (Figure 6G) indicating that remaining USP7 activity might be better exploited by HAdV5 in the H1299 background of HU5 (Figure 6G, compare HC2 + HBX with HU5 + HBX/HBX41108 and HU5 + DMSO).

In conclusion, USP7 probably exerts global positive effects upon adenoviral protein steady-state levels, which become visible when USP7 functions are artificially compromized. As expected, those general decreases in viral protein steady-state levels led to severely reduced progeny virion production, meaning that USP7 plays a pivotal role in adenoviral infection.

### USP7 knockdown and inhibition reduces adenoviral oncogene-mediated cellular transformation efficiency

Together with adenoviral E1A, E1B-55K possesses the ability to transform primary rodent cells [31]. To clarify the potential role of USP7 in cell transformation mediated by adenovirus E1A and especially E1B-55K proteins, we used USP7 specific RNAi (shUSP7) and the USP7 inhibitors HBX and HBX41108 in transformation assays.

**Figure 8:**
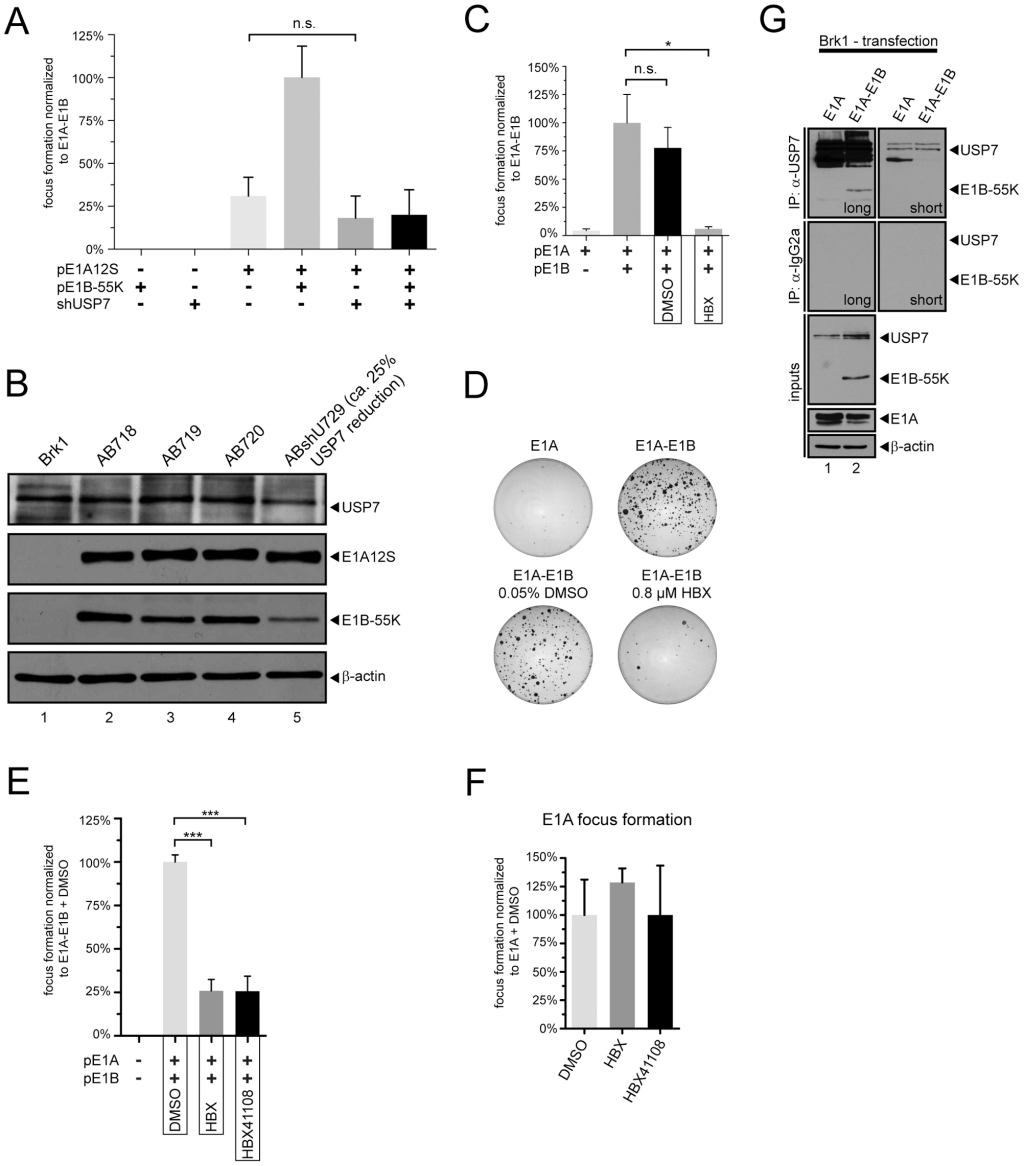
USP7 knockdown and HBX treatment negatively affects cell transformation. (A) Primary baby rat kidney cells were transfected with plasmids encoding E1A12S (pE1A12S), E1B-55K (pE1B-55K) and USP7 shRNAs (shUSP7) as indicated. Morphologically transformed colonies were scored 5 weeks after transfection. Focus forming activity is represented as a percentage of pE1A12S-pE1B-55K (E1A–E1B) activity. The mean and standard deviation are presented for three independent experiments. P-values of unpaired, two-tailed t-tests (n.s. = not significant). (B) Total cell extracts were prepared from Brk1 cells and cell lines derived from transformed foci as indicated and subjected to immunoblotting to detect E1A12S (M73), E1B-55K (2A6), USP7 (6E6), and β-actin (AC-15). (C) Primary baby rat kidney cells were transfected with the whole E1A region (pE1A) or an plasmid containing the E1A plus E1B region (indicated as double positive for pE1A and pE1B) and were treated with DMSO, HBX (ca. 4 days post transfection), or left untreated for several weeks until foci were visible and then scored. The mean and standard deviation are presented for three independent experiments. P-values of unpaired, two-tailed t-tests (*P<0.05, n.s. = not significant). (D) Representative crystal violet stained plates showing foci from each transfection in (C) are shown as an example. (E) Primary Brk cells were transfected as in (C) along with empty vector control (double “−”). As indicated, cells were treated with DMSO, HBX (0.8 µM end concentration) and HBX41108 (0.8 µM end concentration) after first appearance of foci. The mean and standard deviation are presented for three independent experiments. P-values of unpaired, two-tailed t-tests (***P<0.0001). (F) Primary Brk cells were transfected with pE1A and processed as in (E). (G) Primary baby rat kidney (Brk) cell-derived Brk1 cells were transfected as in (C). Cells were harvested 72 h p.t. and subjected to coimmunoprecipitation experiments as in Figure 1E.

Primary baby rat kidney (Brk) cells were transfected with plasmids encoding E1A in combination with E1B-55K and shUSP7 (Figure 8A). Consistent with previous results [32], [55], E1A alone had more restricted focus forming activity, but cotransfecting the cells with E1B-55K expression plasmids increased the number of foci three to four-fold. As expected, in the presence of shUSP7, E1B-55K had little effect in cooperative focus formation, suggesting a strong requirement for USP7 in E1A/E1B-55K-mediated cell transformation. Additionally, shUSP7 had no significant effect on sole E1A-induced focus formation ruling out off-target effects (Figure 8A).

To investigate in detail how USP7 shRNAs might inactivate cell transformation by adenovirus oncogenes, a panel of transformed monoclonal Brk cell lines was established from E1A/E1B-55K (AB), and E1A/E1B/shUSP7 (ABshU) transformed foci. An shUSP7 cotransformed cell line (ABshU729, ca. 25% USP7 kd efficiency; Figure 8B, lane 5) was characterized by immunoblot in comparison to the Brk1 cells (a spontaneously transformed rat cell line derived from primary Brk cells) and reference cell lines transformed with E1A/E1B-55K plus empty vector constructs for shRNAs (AB718–720; Figure 8B, lanes 2–4). In accordance with the transfection data in the USP7 knockdown cell lines, E1B-55K expression was detected in ABshU729 cells, although reduced in comparison to reference AB cells. In all of the established cell lines E1A protein was found to be presented in similar amounts. Thus, it can be concluded that the influence of shUSP7 on the transformation process mainly affects the functions of E1B-55K.

The USP7 inhibitor HBX was applied in similar transformation assays to substantiate the role of USP7 in adenoviral oncogene-mediated cell transformation processes (Figure 8C). Plasmid-based transformation of primary rodent cells with E1A and E1B encoding plasmids was visualized by crystal violet staining of cell foci (representative plates in Figure 8D). Quantification of several experiments revealed a marked reduction in cell foci number upon HBX treatment (Figure 8C) similar to the shRNA experiments (Figure 8A). Interestingly, applying the inhibitor reduced foci formation to that of E1A-induced transformation alone, again suggesting that the effect of HBX treatment was specifically exerted upon E1B-55K. DMSO control-treated cells showed no significant change in foci formation compared to untreated cells (Figure 8C).

To test the ability of another compound against USP7 functions, HBX was applied in parallel with HBX41108. In effect, both compounds exerted almost identical efficacy in reducing the focus forming activity of E1A-E1B indicating similar specificity upon USP7 (Figure 8E). More importantly, application both compounds displayed no further reduction in foci formation activity after sole E1A transfection. This should rule out mere detrimental effects upon cell growth being responsible for reduced foci formation after HBX or HBX41108 treatment (Figure 8F).

It is notable that different to the previous transformation assays with inhibitor application (Figure 8C), in Figure 8E and F inhibitors were applied only after foci were already visible to reduce the overall time of inhibitor incubation. This might explain the lower efficiency in reducing E1A–E1B focus formation activity compared to the assay in Figure 8C.

Since interaction between USP7 and E1B-55K was only shown in transformed human cells, it was necessary to demonstrate that this binding also occurs in transformed rat cells. Indeed, it was possible to coprecipitate E1B-55K from E1B-plasmid transfected Brk1 cells, indirectly implying that this interaction also plays an important role in this setting (Figure 8G, lane 2).

These results clearly demonstrate the important role of USP7 in adenoviral oncogene-mediated transformation processes and show that shRNA or small-molecule inhibitor treatment can efficiently reduce the number of transformed cells in the experimental set-ups.

## Discussion

Since USP7 was first discovered as a herpesviral interacting protein by Meredith and colleagues [26], numerous studies have contributed to our knowledge about this deubiquitinating enzyme (DUB) and defined its important role in not only herpesviral diseases but also cancer-related processes. Until now, four herpesviruses have been described to be associated with USP7, namely Herpes simplex virus type 1 (HSV-1), Epstein-Barr virus (EBV), Kaposi's sarcoma-associated herpesvirus (KSHV) and Human cytomegalovirus (HCMV). Here, to this list we add, for the first time, a virus from the family of *Adenoviridae* and provide evidence that USP7 functions promote efficient adenovirus replication and oncogenicity.

We demonstrate direct association between the adenoviral E1B-55K protein and cellular USP7 by several assays. Functionally, USP7 is very significant for adenoviral replication and oncogene-mediated cellular transformation of Brk cells. Interestingly, not only E1B-55K protein levels seem to be dependent on USP7 functions, but also several other adenoviral proteins, too. For example, E2A protein levels were also diminished upon USP7 knockdown and partly after inhibitor application (Figure 6 and S5). Moreover, E2A colocalized with USP7 indicating USP7 being relocalized to adenoviral replication centers (Figure 2) and also providing a possibility of E2A-mediated USP7 relocalization since E1B-55K seems to be dispensable for this phenotype. In a functionally different context, this can have implications in gene expression control like it has been shown for USP7 in its activities upon EBNA1 (facilitating binding of EBNA1 to latent viral genome *oriP* elements) and p53 (supporting sequence-specific DNA binding activity) [28]. Therefore, it can be speculated that USP7 exerts similar functions on E2A. Nevertheless, it should be considered that just three viral and three cellular elements have been found to be sufficient for efficient adenovirus DNA replication *in vitro*: Adenovirus DNA-polymerase, precursor- or preterminal protein (pTP), E2A (also called DNA binding protein [DBP]), cellular NFI, NFII, and topoisomerase I [56]. In this respect, USP7 would have supportive rather than essential functions, and we are now investigating possible roles of USP7 for E2A activity.

### USP7 knockdown or inhibition leads to similar negative effects on adenoviral replication like USP7 knockout

Many efforts have been invested to find new drugs against DUBs or other proteins related to the ubiquitin-proteasome system (UPS). Aberrantly regulated DUBs are described to be involved in specific human diseases such as cancer and neurodegenerative disorders [57]. Since USP7 is as yet the only DUB discovered to be directly connected to both cancer and infectious diseases, it is very enticing to find suitable inhibitors that can be used efficiently and specifically against USP7. In a patent from 2006, Hybrigenics described several cyano-indenopyrazine substances that exerted functional inhibition of USP7 [47]. One of these Hybrigenics substances was resynthesized (due to the lack of commercially available inhibitors; here called HBX) and used in this study to perform inhibitor assays on adenovirus-infected cells in order to investigate the functional consequences of the USP7-E1B-55K interaction and prove that USP7 inhibition, like USP7 knockdown or knockout, can efficiently reduce virus replication. In the course of our studies, a derivative of HBX, HBX41108, was released and this compound was also implemented in our assays to compare efficacy of both compounds supporting specificity upon USP7 in diverse assays of this study.

Both USP7 knockdown and inhibitor application severely reduced E1B-55K protein levels, and in the knockdown setting as well as after USP7 inhibitor application the half-life of E1B-55K was significantly shorter (Figure S4A and S4B). In trying to reveal the precise mechanism underlying USP7-mediated E1B-55K stabilization, we invested much effort in demonstrating first, ubiquitination of E1B-55K and second, subsequent deubiquitination by USP7. Unfortunately, our efforts were not successful. As for now, there is no report that has investigated possible ubiquitination of E1B-55K which also involves identification of the respective E3 ligase. So, only after clarifying these two questions deubiquitination by USP7 can be tackled. Moreover, it is far from clear that stabilization of E1B-55K is mediated through deubiquitination and degradation by ubiquitination, although some indications may lead to that assumption and our study might support this theory. However, considering known functions of USP7 in gene expression control through regulation of histone proteins and the known relationship between adenoviral gene expression activity and the chromatinization of the adenoviral genome inside the nucleus of infected cells, it is also likely that USP7-mediated E1B-55K stability might be exerted through a mechanism other than direct deubiquitination of E1B-55K [58]–[60].

In keeping with the functions exerted by E1B-55K in the adenoviral life cycle, many defects in virus replication, such as reduced late protein production, can be explained by decreased functionality of this protein and the complexes it forms during productive replication. For example, late adenoviral mRNA transport is carried out by a complex comprising E1B-55K and E4orf6 [61]–[66]. As a result of reduced complex formation, it is very likely that these mRNA species do not accumulate sufficiently, which is eventually reflected by lower adenoviral capsid protein production (Figure 6 and S5).

However, during the course of this study, it became clear that USP7 not only specifically targets E1B-55K, but also exerts positive effects on other early proteins (and late proteins). To our surprise E1A and E2A steady-state protein levels were also negatively affected by USP7 knockdown and inhibition. However, certain differences between knockdown and inhibition were observed. For instance, E1A protein levels are clearly lower in knockdown *versus* the control cell line (Figure 6A and S5A), but showed no differences or even increased after inhibitor treatment depending on the cell type treated (Figure 6B and S5C). While it remains enigmatic why increased E1A levels do not lead to higher protein levels of those genes regulated by early viral promoters (e.g. E2A, Figure S5C), it is much easier to understand that lower E1A protein levels lead to reduced activation of viral early promoters, with a subsequent delay in protein expression of the respective genes.

Interestingly, Fessler and Young demonstrated that lowered expression from the major late promoter (MLP) leads to increases in the expression of early genes among them E1A and E1B (the mechanism of this phenomenon has not been clarified in detail yet) especially when MLP expression is hampered at late times of infection [67]. This might explain the contrast between USP7 knockdown and inhibition in relation to the E1A and E1B-55K reduction (compare Figure 6A and S5A with Figure 6B and S5C). Knockdown is a permanent condition in our assays leading to the assumption that USP7 is needed for proper E1A expression in the initial phase of the adenoviral replication cycle. However, inhibitor treatment in our assays starts earliest 9 h p.i. and compromising late gene expression/late protein stability at this time point through USP7 inhibition rather increases than decreases E1A levels and might also explain why E1B-55K reduction is attenuated in contrast to the single transfection assays. But how can USP7 affect late gene expression? Taking into account that USP7 not only affects E1B-55K but also, for example, E2A levels it is probable that E2A functions in promoting DNA replication might be hampered which in turn lead to the observed negative effect on late gene expression [67], [68].

Another aspect to consider, while knockdown affects protein levels *in toto*, inhibitor treatment might affect only one function of a protein without affecting other functions at all. Due to USP7's multi-domain structure, its functions are not only carried out by the enzymatic domain [36], [69]. However, the specific effects of both approaches (knockdown and inhibition) upon other viral proteins such as E1B-55K or structural proteins clearly suggest that USP7 functions are necessary during the whole course of infection. In this context, USP7, having enzymatic activity, represents a potent target for small-molecule inhibitors. Our results clearly indicate that adenoviral progeny virions can be reduced in a significant manner (up to over 90%) even after an established infection using an inhibitor of USP7, results that could, at least qualitatively, also be confirmed with RNAi experiments.

### USP7 in adenoviral transformation processes

Strikingly, both approaches to disrupting USP7 functions also diminished the ability of adenoviral oncogenes to induce cellular transformation. It is not sure but possible that shUSP7 induced low levels of E1B-55K which might explain the fewer counted cell foci in this set-up (Figure 8B). Similarly, addition of the USP7 inhibitors HBX (Figure 8C and E) and HBX41108 (Figure 8E) also lowered the number of transformed cell foci.

Considering that USP7 is already known to be involved in tumor pathways, the observed phenotypes may be explained by two possible scenarios: First, p53 is activated, accumulates and promotes antiproliferative activities due to the instability of its negative regulator Mdm2. It has been shown that Mdm2 is the primary target of USP7-mediated stabilization. Thus, inhibition of USP7 in this setting might lead to reduced Mdm2 levels. Second, increased Daxx proapoptotic activity supports cell death. As in the case of p53, Daxx levels increase due to missing negative Mdm2 regulation after USP7 inhibition. Additionally, in transformation settings, Daxx functions are antagonized by E1B-55K, and as shown in Figure 5 E1B-55K levels definitely depend on functional USP7. Indeed, it was possible to demonstrate an E1B-55K-USP7 interaction in rat (equivalent to human) cells (Figure 8G), indirectly supporting a direct relationship between USP7 and E1A-E1B-55K-mediated cellular transformation. Therefore, similar USP7-dependent mechanisms may play an important role during adenoviral lytic infection and adenoviral-oncogene-mediated transformation processes, emphasizing the extraordinary relationship between USP7 and E1B-55K.

In summary, for the first time, one cellular protein can be linked to efficiently reducing adenovirus yield and virus-mediated cellular transformation. Therefore blocking the activity of USP7 could potentially be used to treat adenovirus infections. Particularly, pediatric patients undergoing allogenic stem cell transplantation are vulnerable to disseminated adenovirus infections, leading to a high mortality rate [11]. Hence, there is a need for potent antiviral therapeutics against adenoviruses that allow suppression of the virus at different stages in the replication cycle [12]. USP7 represents a striking drug target.

## Materials and Methods

### Cell culture, virus infection and transfection

A549 and H1299 cells were cultivated as described [70]. HCT116 and HCT116 USP7 double-knockout cells (USP7 −/− [KO]; kind gifts of Dr. Bert Vogelstein) were grown in McCoy's 5a Medium (GIBCO) supplemented with 10% or 15% (USP7 KO) fetal bovine serum (FBS, PAA), 100 U of penicillin, and 100 µg of streptomycin per ml. Primary Brk (baby rat kidney) cells and the Brk-derived cell line Brk1 [31] were grown in Dulbecco's modified Eagle medium (DMEM, PAA) supplemented with 5 to 10% FBS, 100 U of penicillin, and 100 µg of streptomycin per ml in a 5% CO_2_ atmosphere at 37°C. APU5 and APU6 (USP7 knockdown, kd) cells were grown under the same conditions as Brk cells, and HC2 and HU5 (USP7 kd) cells were grown under the same conditions as H1299 cells. Additionally, all the knockdown and control cell lines were also grown under constant puromycin challenge (2 µg/ml, Calbiochem). Infections with wt (H5*pg*4100) and E1B minus (H5*pm*4149) adenoviruses and subsequent virus yield experiments were carried out as described earlier [70]. Infection with E1B minus2 (*dl*1520) was carried out like described earlier [39]. Virus yield was calculated as described earlier [70] in virus particles per cell (FFU/cell), these results were normalized and presented as a function of untreated or control cells. This calculation allowed negative cell growth effects exerted by HBX or HBX41108 to be neglected. In indicated experiments HBX, HBX41108 and DMSO was added 15 hours before cell harvest at denoted concentrations or incubated for 24 hours. Transfections with plasmid DNAs used PEI as a transfecting agent and were carried out as described earlier [70]. Other experiments included cycloheximide addition (SIGMA) and harvest at indicated time points (end concentration 10 µM). The USP7 inhibitor HBX (example 1) was synthesized as described earlier [47].

### Indirect immunofluorescence and software tools

Indirect immunofluorescence staining and image capturing was carried out as described earlier [70]. Processing and layout of images were accomplished using Adobe Photoshop and Illustrator CS4 software tools. Statistical analyses were all performed with Microsoft Excel 2007 and GraphPad Prism 5. Western blot band intensities were analyzed with ImageJ 1.45s.

### Co-IP assays, protein analyses and antibodies

Coimmunoprecipitation (Co-IP) assays were performed with the anti-USP7 (3D8 or 6E6) and, as a control, a non-specific IgG2a monoclonal rat antibody (1–2 µg/sample) was used. Protein analyses, Western blots and antibody usage in general were also carried out as described earlier [70]. The USP7 antibodies 3D8 and 6E6 were used for Western blots as a 1∶10 dilution in phosphate-buffered saline (PBS) containing 0.05% Tween 20 (AppliChem) and 1% nonfat dry milk. Following steps as referenced above.

### FACS-FRET

2.5×10^5^-1.0×10^6^ H1299 cells were seeded into 6-well plate wells or 10 cm dishes (Sarstedt) transfected with pECFP-C1, pEYFP-C1, pEYFP-ECFP (pEYFP-ECFP is a fusion protein; the first three plasmid constructs were kind gifts from Dr. Carina Banning), YFP-E1B-55K (YFP-55K) and USP7-CFP in different combinations as indicated. 24–48 hours post transfection cells were harvested and assayed. Following steps were performed as previously described [37], [71].

### Expression, purification of recombinant fusion proteins and GST pull-down

The GST fusion proteins E1B-55K, E1B 156R, E1B 93R, E1B 83–188, E1B 93R 1–82, E1B 1–162, USP7 TD, USP7 CD, USP7 C1 and USP7 C2 were expressed and purified as described earlier [70]. For the GST pull-down assays equal amounts of fusion proteins were incubated with a defined quantity of cell lysate. This mixture was then incubated for 2 h at 4°C on a turning rotor. The proteins bound to the Glutathione Sepharose (GE Healthcare) were subsequently precipitated by centrifugation (6500 rpm, 5 min, 4°C), six times washed with PBS or lysis buffer, centrifuged and boiled in 25 µl of SDS sample buffer. The protein samples were then analyzed by SDS-PAGE and Western blotting. Input of recombinant proteins was analyzed by Coomassie brilliant blue staining (CB).

### MTS-based proliferation assay

1.5×10^3^ cells were seeded per 96-well plate well (Falcon). 12–20 hours later, culture medium with different compound concentrations (concentration series in triplicates) was added to cells, replacing the old medium. As controls, untreated and compound solvent (DMSO) treated cells were used. For all compound and solvent treated cells, the final concentration of DMSO (usually 0.05%) was equal. Cells were incubated for different time points with compound, usually 24, 48 and 72 h and then cell proliferation was measured with the Promega CellTiter 96 Aqueous One Solution Cell Prolifertation Assay (MTS = (3-(4,5-dimethylthiazol-2-yl)-5-(3-carboxymethoxyphenyl)-2-(4-sulfophenyl)-2H-tetrazolium)) according to manufacturer's instructions. The resulting color reaction was measured with a plate reader at 490 nm (BioTek SynergyMx).

### Transformation assays

Transformation assays were carried out as described earlier [72], [73]. In addition, the USP7 inhibitor HBX and DMSO were also included in the growth medium as indicated. Transformation assay in Figure 8C was carried out with inhibitor addition 4–6 days after transfection. Transformation assay in Figure 8E and F were carried out with inhibitor addition after first foci were visible.

### Generation of cell lines

To establish stable rat cell lines, foci were isolated using a glass cloning cylinder (5 mm diameter) circling single colonies. The cells within the cylinders were trypsinized with 100 µl of trypsin/EDTA solution. When the cells were detached from the dish, they were transferred into the wells of a 24-well plate (Falcon) containing DMEM with 10% FBS. These cells were grown for several weeks and expanded to obtain monoclonal cell lines.To establish stable monoclonal USP7 knockdown cell lines from A549 and H1299, these cells were seeded onto 6-well dishes and transfected with pSuper-shUSP7 (shUSP7) or empty vector using PEI. One day after transfection, fresh media containing 2–3 µg/ml of puromycin (Sigma) was added to the transfected cells. Three days later, the cells were split in a ratio of 1∶30, and seeded onto two 150 mm-diameter tissue culture dishes (Falcon). Fresh media containing puromycin were added to the cells every 3–4 days to select the stably transfected ones. Three weeks after splitting, foci were chosen and isolated as above to establish monoclonal cell lines and expanded. Puromycin was always included in the growth medium of these cells. HC2 contains the pSuper.retro.puro empty vector and HU5 is stably transfected with shUSP7. APU5 and APU6 are both stably transfected with shUSP7 whereas USP7 knockdown is only detected in APU6.

### NCBI accession numbers

HAdV5 E1B-55K protein: AP_000199.1. Human USP7 (HAUSP) protein: NP_003461.2.

## Supporting Information

Figure S1 **Yeast two-hybrid screen analysis.** (A) Yeast cells were transformed with the plasmid construct E1B-DBD (DNA binding domain fused to a HAdV5 E1B-55K truncation [first 262 amino acids]). First selection of positive clones was performed on tryptophane-negative plates (W^−^). Next, positive yeast clones were subjected to a second transformation with a human cDNA gene bank from EBV-transformed human peripheral lymphocytes (coding fusions with activation domain, AD). Subsequent second selection was carried out on histidine-, tryptophane-, leucine-negative plates. Fully active reporter gene functions were assayed in a filter-lift-assay to select for β-galactosidase activity and possible “hits”. No activity was found after sole transformation of E1B-DBD. Two positive clones (blue/greenish colony) were processed for DNA extraction and subsequent sequencing. Identification of the obtained DNA sequence was performed using the NCBI BLAST tool and revealed N-terminal DNA fragments of USP7. Red bars represent length and position of the identified DNA fragments in comparison to the whole USP7 nucleotide sequence. (B) Sequences of USP7 DNA fragment 1 and 2 from positive yeast clones after yeast two-hybrid screen.
(TIF)

Figure S2 **USP7 is relocalized during infection with an adenovirus lacking E1B functions.** H1299 cells were infected at an MOI of 20 FFU/cell with wt (H5*pg*4100; panels a to C) and E1B minus2 (*dl*1520; panels D to F) and 24 h p.i. analyzed by *in situ* immunofluorescence staining for E2A (B6-8; section B and E), and USP7 (3D8; section C and F). The overlays (merge) of the green and red images are shown in A and D.
(TIF)

Figure S3 **Dose-response curves of different cell lines upon USP7 inhibitor HBX treatment.** (A–C) A549, H1299 and Brk1 cells were seeded into 96-well plates (1.5×10^3^/well). Treatment of cells with a series of HBX concentrations was performed for 24, 48, 72 h or cells were treated with DMSO or left untreated (ctrl). S.e.m. values from a minimum of three independent experiments. Plate reader read-out was performed at 490 nm.
(TIF)

Figure S4 **Knockdown or inhibition of USP7 results in higher E1B-55K turnover.** (A) APU5 and APU6 cells were infected at an MOI of 20 FFU/cell with wt virus (H5*pg*4100). 24 hours after infection, cells were treated with cycloheximide (CHX, 10 µM end concentration). The cells were harvested at different time points after treatment as indicated. Total cell extracts were prepared and subjected to immunoblotting by 3D8 antibody detecting USP7, 2A6 antibody detecting E1B-55K, and AC-15 detecting β-actin. (B) A549 cells were infected at an MOI of 20 FFU/cell with wt virus (H5*pg*4100). 15 hours after infection, cells were treated with CHX as in (A) plus HBX, HBX41108 or DMSO. As a control, cells were treated with HBX, HBX41108 or DMSO without addition of cycloheximide as indicated. (C) Represented are immunoblot stainings (steady-states/loading controls) of (B) for the indicated proteins.
(TIF)

Figure S5 **USP7 knockdown or inhibition has negative effects on adenoviral proteins and virus growth.** (A) HC2 and HU5 cells were infected with wt virus (MOI 20 FFU/cell). Total cell extracts were prepared at indicated h p.i. and subjected to IB to detect USP7, β-actin, E1A, E1B-55K, E2A, L4-100K and viral late proteins (Roman numerals). ImageJ analysis of the USP7 Western blots was performed to determine the band intensity reduction in HC2 compared to HU5 cells. (B) The USP7 knockdown cell line HU5, and its corresponding control cell line HC2 (permanently transfected with empty vector) were infected with wt virus (MOI 20 FFU/cell). Viral particles were harvested at 24 and 48 h p.i. and infectious virus particles produced were determined by quantitative E2A staining on HEK293 cells. Virus production is represented as a percentage of virus production in the respective control HC2 cells. The results represent the averages of at least five independent experiments. Error bars indicate the standard error of the mean. P-values of unpaired, two-tailed t-tests (*P<0.05, **P<0.01). (C) H1299 cells were infected as in (A) but harvested at 24 and 48 h p.i. and analyzed to detect indicated proteins. 15 h before cells were harvested, DMSO or HBX was added to the cells at indicated concentrations. (D) A549 cells were treated as in (C) except without adding virus particles. (E) Same procedure as in (C), but here virus was extracted at indicated time points. The virus yield was determined by quantitative E2A immunofluorescence staining on HEK293 cells. The results represent the average of at least three independent experiments. Bars indicate the standard error of the mean values. Virus yield efficiency is represented as a percentage of untreated wt infected A549 cells. P-values of unpaired, two-tailed t-tests (**P<0.01, ***P<0.001, n.s. = not significant).
(TIF)

Figure S6 **USP7 inhibitor HBX reduces the amount of E1B-positive cells.** (A to D) A549 and H1299 cells were infected with wt virus (H5*pg*4100) (MOI = 20 FFU/cell) and analyzed by *in situ* immunofluorescence staining for E1B-55K (2A6) and USP7 (3D8). Additionally, cells were subjected to DMSO or HBX treatment as described in Figure 6B and S5C. E1B-positive cells were quantified and normalized to total cell number. S.e.m. of at least three experiments. P-values of unpaired, two-tailed t-tests (*P<0.05, ***P<0.001, n.s. = not significant). Border of nuclei are represented by dotted lines. White bars represent 10 µm length.
(TIF)

Text S1 **The supporting information contains a list of all antibodies used in this study and the corresponding references.**
(DOC)
