# Supplementary figures and images for: A Ubiquitin-specific Protease Possesses a Decisive Role for Adenovirus Replication and Oncogene-mediated Transformation
Source: PLoS Pathog. 2013 Mar 28;9(3):e1003273. doi: 10.1371/journal.ppat.1003273 (PMC3610741; doi:10.1371/journal.ppat.1003273)

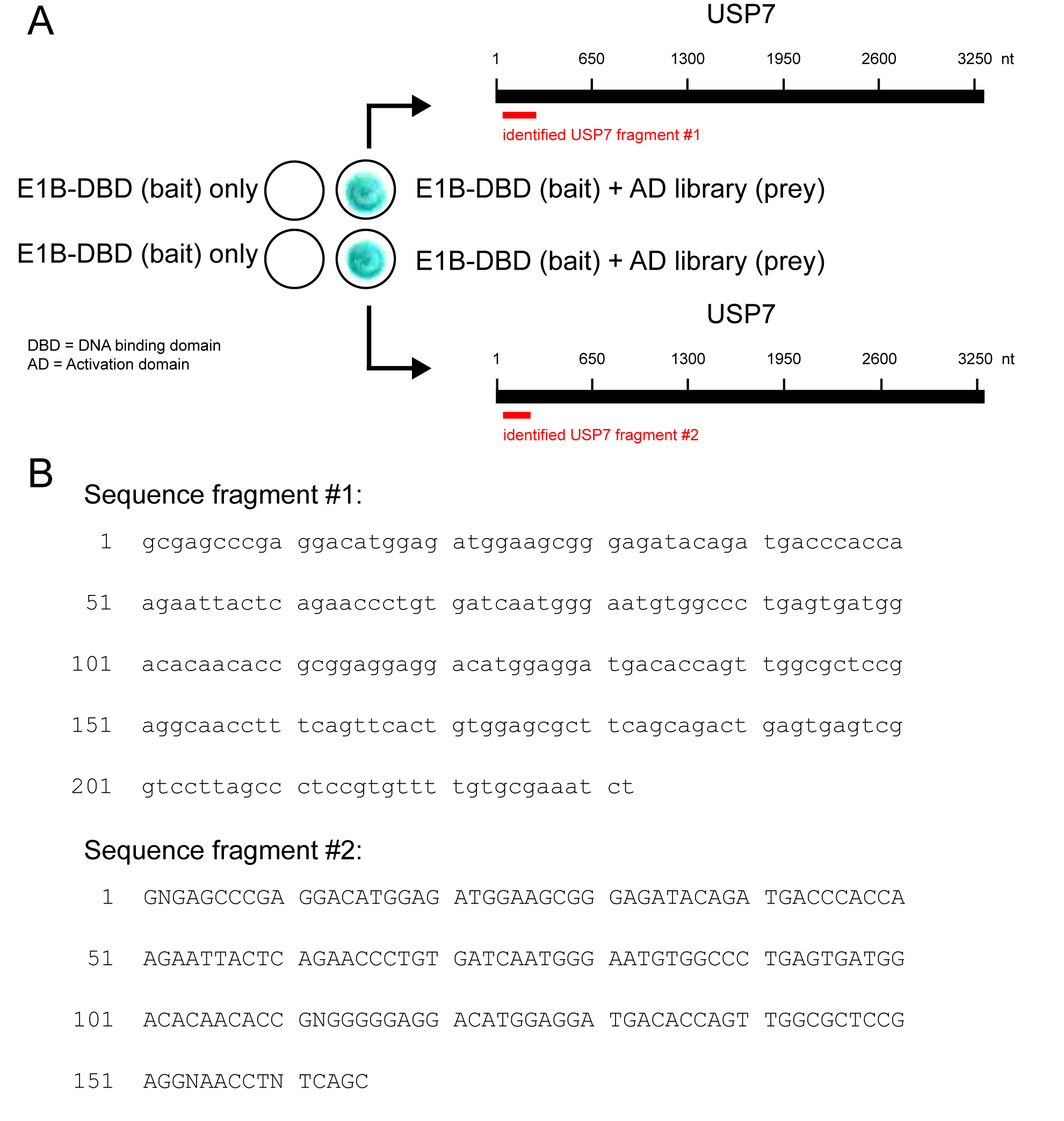

Supplement: Figure S1 — Yeast two-hybrid screen analysis. (A) Yeast cells were transformed with the plasmid construct E1B-DBD (DNA binding domain fused to a HAdV5 E1B-55K truncation [first 262 amino acids]). First selection of positive clones was performed on tryptophane-negative plates (W−). Next, positive yeast clones were subjected to a second transformation with a human cDNA gene bank from EBV-transformed human peripheral lymphocytes (coding fusions with activation domain, AD). Subsequent second selection was carried out on histidine-, tryptophane-, leucine-negative plates. Fully active reporter gene functions were assayed in a filter-lift-assay to select for β-galactosidase activity and possible “hits”. No activity was found after sole transformation of E1B-DBD. Two positive clones (blue/greenish colony) were processed for DNA extraction and subsequent sequencing. Identification of the obtained DNA sequence was performed using the NCBI BLAST tool and revealed N-terminal DNA fragments of USP7. Red bars represent length and position of the identified DNA fragments in comparison to the whole USP7 nucleotide sequence. (B) Sequences of USP7 DNA fragment 1 and 2 from positive yeast clones after yeast two-hybrid screen. (TIF) [file ppat.1003273.s001.tif]

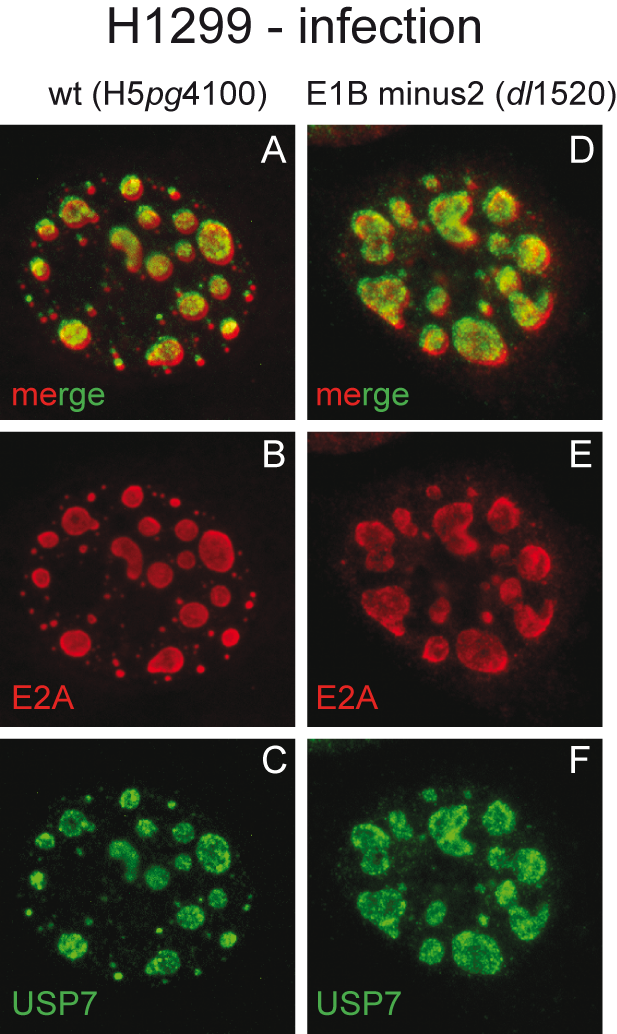

Supplement: Figure S2 — USP7 is relocalized during infection with an adenovirus lacking E1B functions. H1299 cells were infected at an MOI of 20 FFU/cell with wt (H5pg4100; panels a to C) and E1B minus2 (dl1520; panels D to F) and 24 h p.i. analyzed by in situ immunofluorescence staining for E2A (B6-8; section B and E), and USP7 (3D8; section C and F). The overlays (merge) of the green and red images are shown in A and D. (TIF) [file ppat.1003273.s002.tif]

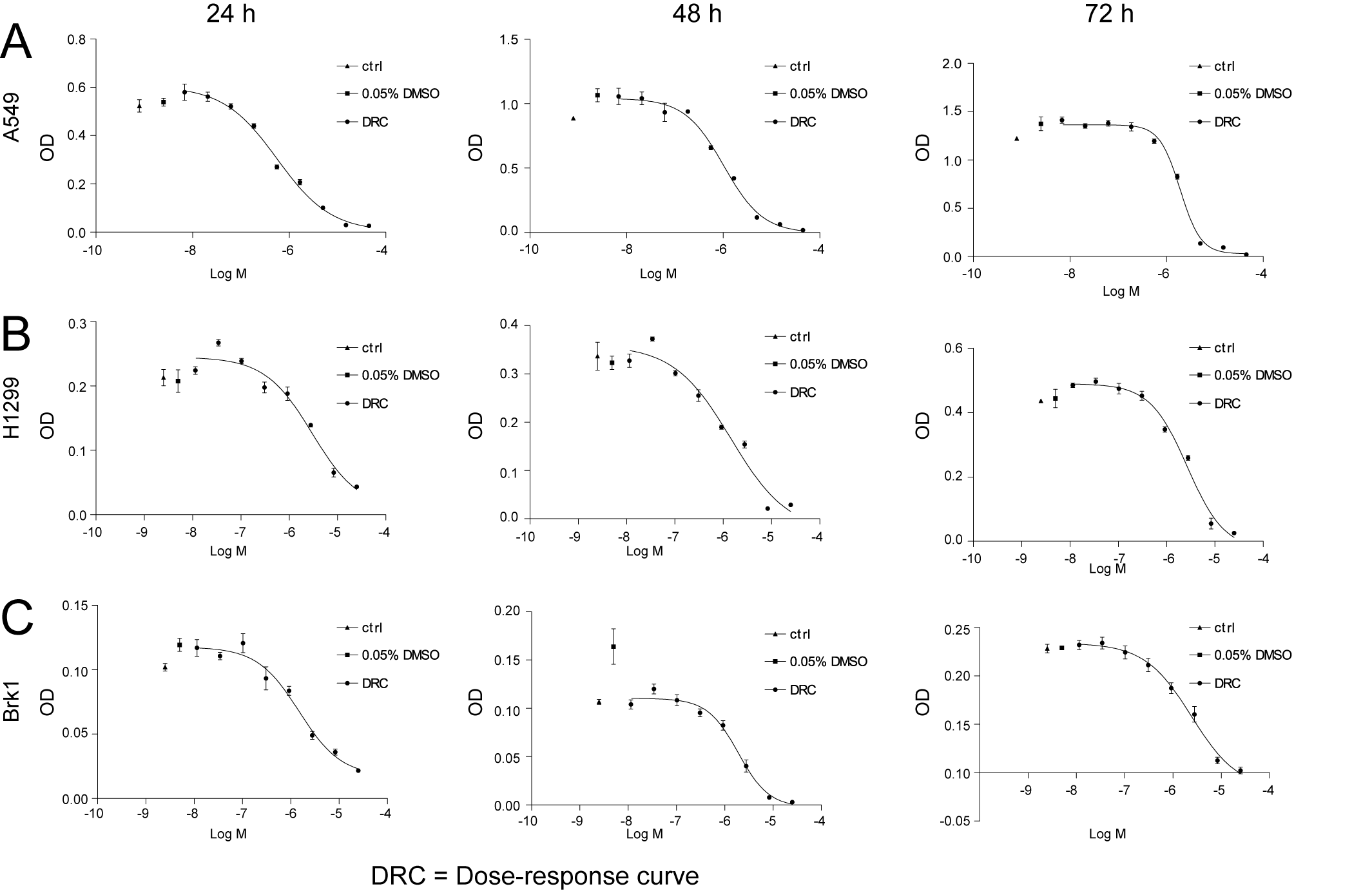

Supplement: Figure S3 — Dose-response curves of different cell lines upon USP7 inhibitor HBX treatment. (A–C) A549, H1299 and Brk1 cells were seeded into 96-well plates (1.5×10^3^/well). Treatment of cells with a series of HBX concentrations was performed for 24, 48, 72 h or cells were treated with DMSO or left untreated (ctrl). S.e.m. values from a minimum of three independent experiments. Plate reader read-out was performed at 490 nm. (TIF) [file ppat.1003273.s003.tif]

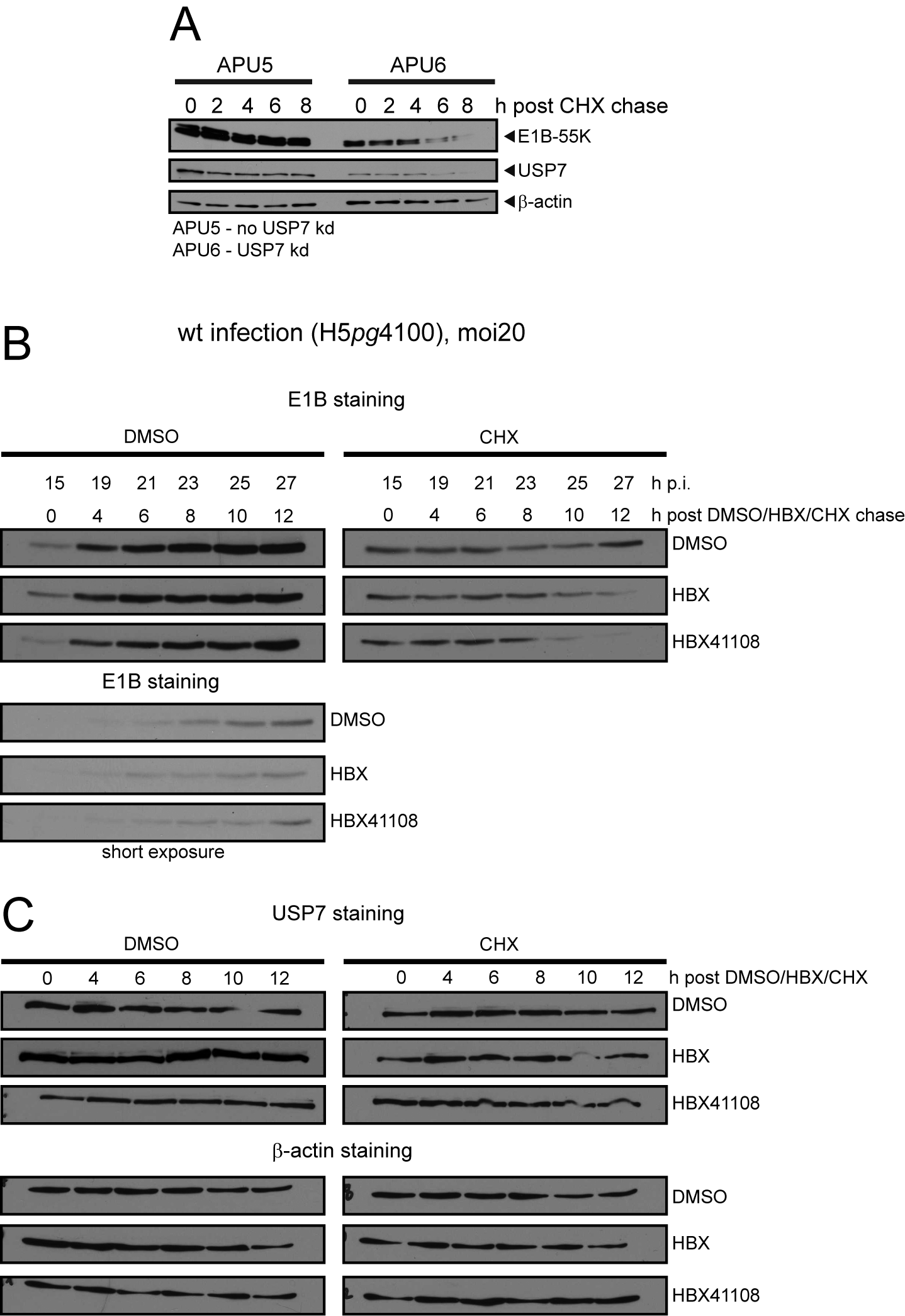

Supplement: Figure S4 — Knockdown or inhibition of USP7 results in higher E1B-55K turnover. (A) APU5 and APU6 cells were infected at an MOI of 20 FFU/cell with wt virus (H5pg4100). 24 hours after infection, cells were treated with cycloheximide (CHX, 10 µM end concentration). The cells were harvested at different time points after treatment as indicated. Total cell extracts were prepared and subjected to immunoblotting by 3D8 antibody detecting USP7, 2A6 antibody detecting E1B-55K, and AC-15 detecting β-actin. (B) A549 cells were infected at an MOI of 20 FFU/cell with wt virus (H5pg4100). 15 hours after infection, cells were treated with CHX as in (A) plus HBX, HBX41108 or DMSO. As a control, cells were treated with HBX, HBX41108 or DMSO without addition of cycloheximide as indicated. (C) Represented are immunoblot stainings (steady-states/loading controls) of (B) for the indicated proteins. (TIF) [file ppat.1003273.s004.tif]

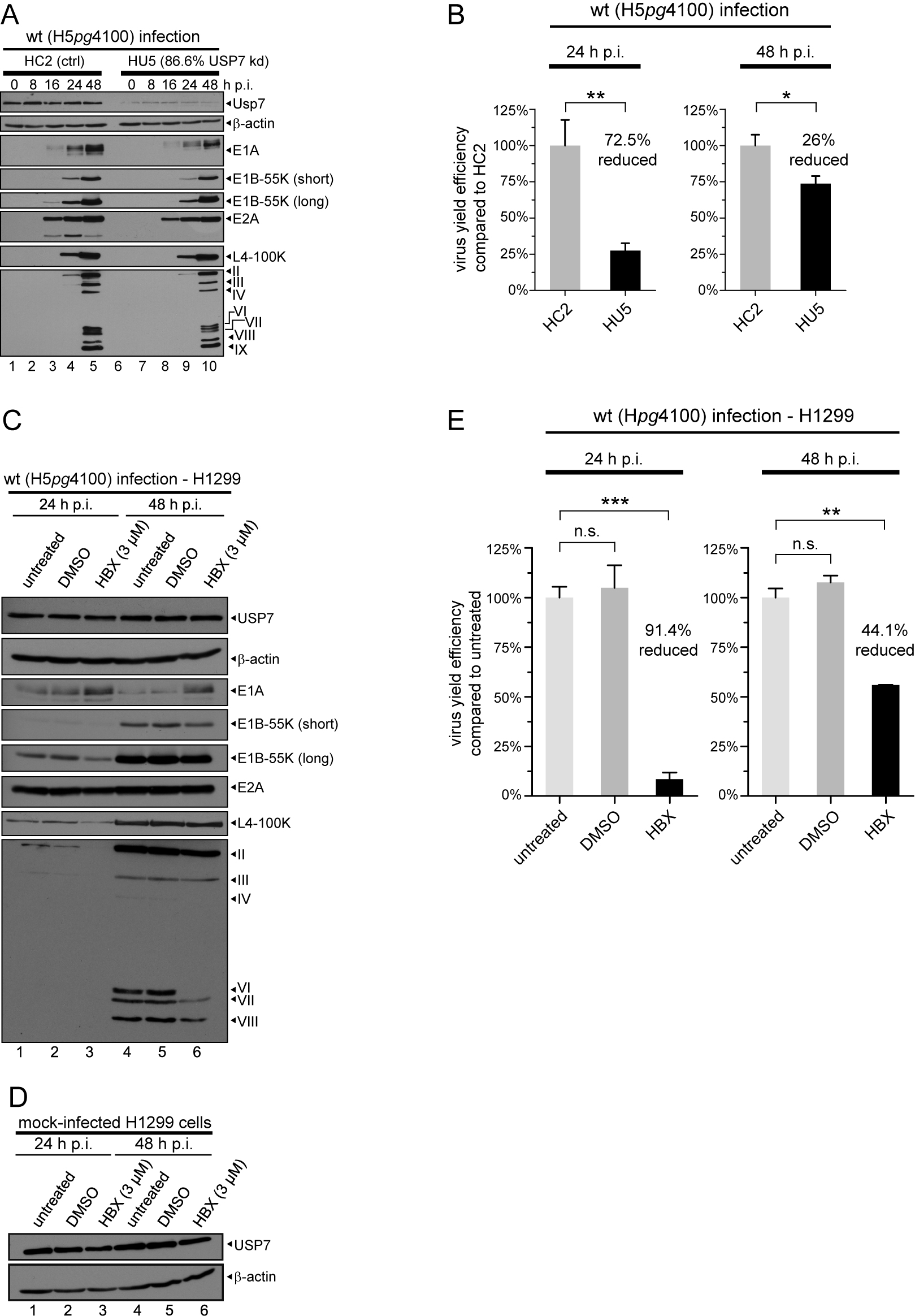

Supplement: Figure S5 — USP7 knockdown or inhibition has negative effects on adenoviral proteins and virus growth. (A) HC2 and HU5 cells were infected with wt virus (MOI 20 FFU/cell). Total cell extracts were prepared at indicated h p.i. and subjected to IB to detect USP7, β-actin, E1A, E1B-55K, E2A, L4-100K and viral late proteins (Roman numerals). ImageJ analysis of the USP7 Western blots was performed to determine the band intensity reduction in HC2 compared to HU5 cells. (B) The USP7 knockdown cell line HU5, and its corresponding control cell line HC2 (permanently transfected with empty vector) were infected with wt virus (MOI 20 FFU/cell). Viral particles were harvested at 24 and 48 h p.i. and infectious virus particles produced were determined by quantitative E2A staining on HEK293 cells. Virus production is represented as a percentage of virus production in the respective control HC2 cells. The results represent the averages of at least five independent experiments. Error bars indicate the standard error of the mean. P-values of unpaired, two-tailed t-tests (*P<0.05, **P<0.01). (C) H1299 cells were infected as in (A) but harvested at 24 and 48 h p.i. and analyzed to detect indicated proteins. 15 h before cells were harvested, DMSO or HBX was added to the cells at indicated concentrations. (D) A549 cells were treated as in (C) except without adding virus particles. (E) Same procedure as in (C), but here virus was extracted at indicated time points. The virus yield was determined by quantitative E2A immunofluorescence staining on HEK293 cells. The results represent the average of at least three independent experiments. Bars indicate the standard error of the mean values. Virus yield efficiency is represented as a percentage of untreated wt infected A549 cells. P-values of unpaired, two-tailed t-tests (**P<0.01, ***P<0.001, n.s. = not significant). (TIF) [file ppat.1003273.s005.tif]

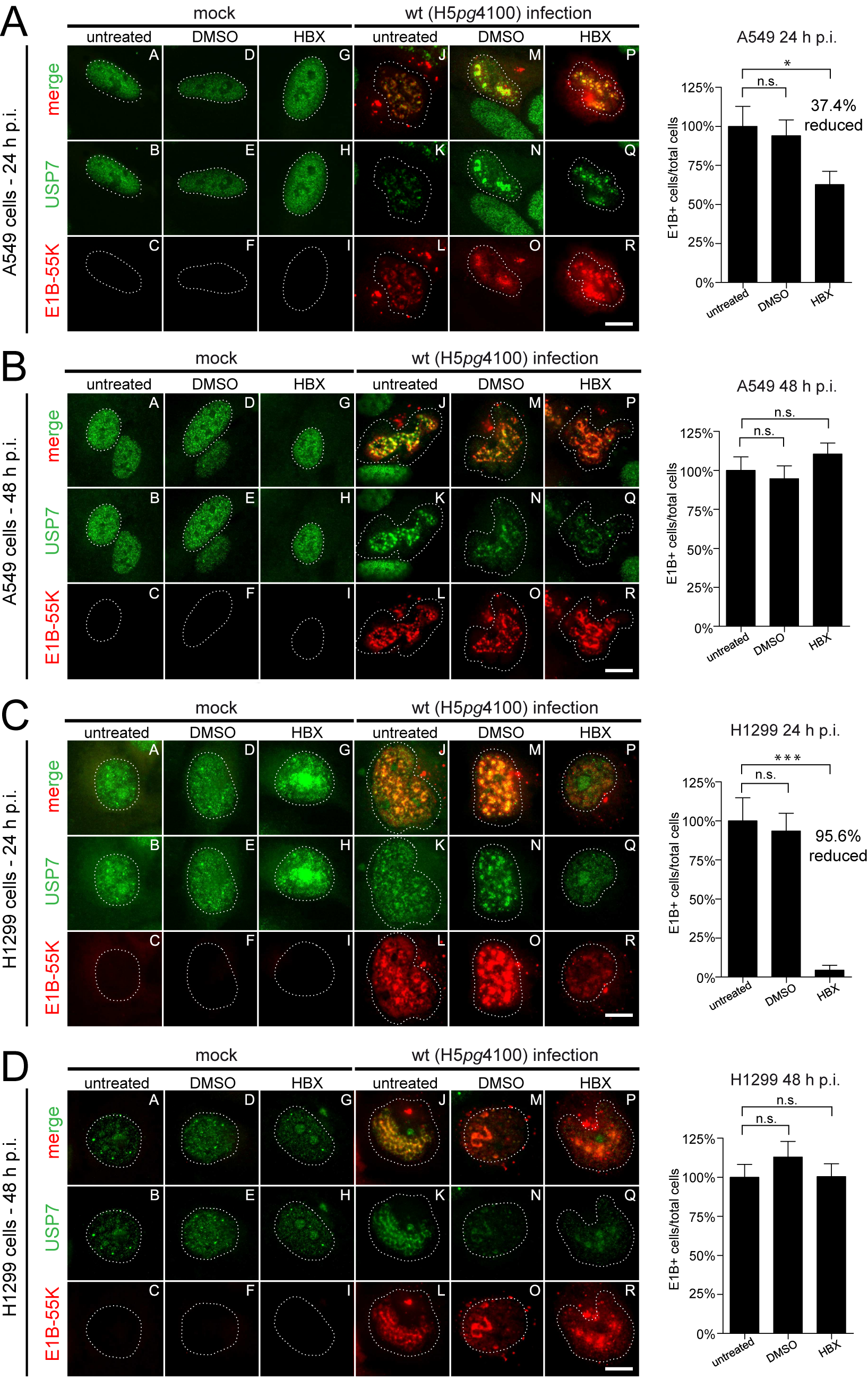

Supplement: Figure S6 — USP7 inhibitor HBX reduces the amount of E1B-positive cells. (A to D) A549 and H1299 cells were infected with wt virus (H5pg4100) (MOI = 20 FFU/cell) and analyzed by in situ immunofluorescence staining for E1B-55K (2A6) and USP7 (3D8). Additionally, cells were subjected to DMSO or HBX treatment as described in Figure 6B and S5C. E1B-positive cells were quantified and normalized to total cell number. S.e.m. of at least three experiments. P-values of unpaired, two-tailed t-tests (*P<0.05, ***P<0.001, n.s. = not significant). Border of nuclei are represented by dotted lines. White bars represent 10 µm length. (TIF) [file ppat.1003273.s006.tif]
