# Supplementary material for: A Ubiquitin-specific Protease Possesses a Decisive Role for Adenovirus Replication and Oncogene-mediated Transformation
Source: PLoS Pathog. 2013 Mar 28;9(3):e1003273. doi: 10.1371/journal.ppat.1003273 (PMC3610741; doi:10.1371/journal.ppat.1003273)
Supplement: Text S1 — The supporting information contains a list of all antibodies used in this study and the corresponding references. (DOC) [file ppat.1003273.s007.doc]

**Antibodies**

Primary antibodies (Ab) against viral factors used in this study included E1A mouse monoclonal Ab (MAb) M73 [1], E1B-55K mouse MAb 2A6 [2], E2A-72K mouse MAb B6-8 [3], L4-100K rat MAb 6B-10 [4], and HAdV5 rabbit polyclonal serum L133 [5]. Primary Ab against cellular proteins included USP7 rat MAb 3D8 and 6E6, Mdm2 (Hdm2) rabbit PAb AF1244 (R&D Systems) and β-actin mouse MAb AC-15 (Sigma-Aldrich, Inc.). Other primary antibodies: GFP/YFP rabbit PAb (sc8334). IgG2a rat MAb (abcam, ab18450) was used as a control antibody for coimmunoprecipitation assays. Secondary Ab conjugated to horseradish peroxidase (HRP) were from Jackson/Dianova. Secondary Abs used in immunofluorescence were from Invitrogen (FITC- or Texas Red-conjugated).

**Supplemental references**

1. Harlow E, Franza BR, Jr., Schley C (1985) Monoclonal antibodies specific for adenovirus early region 1A proteins: extensive heterogeneity in early region 1A products. J Virol 55: 533-546.

2. Sarnow P, Sullivan CA, Levine AJ (1982) A monoclonal antibody detecting the adenovirus type 5-E1b-58Kd tumor antigen: characterization of the E1b-58Kd tumor antigen in adenovirus-infected and -transformed cells. Virology 120: 510-517.

3. Reich NC, Sarnow P, Duprey E, Levine AJ (1983) Monoclonal antibodies which recognize native and denatured forms of the adenovirus DNA-binding protein. Virology 128:480-484.

4. Kzhyshkowska J, Kremmer E, Hofmann M, Wolf H, Dobner T (2004) Protein arginine methylation during lytic adenovirus infection. Biochem J 383: 259-265.

5. Kindsmüller K, Groitl P, Härtl B, Blanchette P, Hauber J, et al. (2007) Intranuclear targeting and nuclear export of the adenovirus E1B-55K protein are regulated by SUMO1 conjugation. Proc Natl Acad Sci USA 104: 6684–6689.
